# Data-Driven Risk Assessment from Small Scale Epidemics: Estimation and Model Choice for Spatio-Temporal Data with Application to a Classical Swine Fever Outbreak

**DOI:** 10.3389/fvets.2017.00016

**Published:** 2017-02-28

**Authors:** Kokouvi Gamado, Glenn Marion, Thibaud Porphyre

**Affiliations:** ^1^Biomathematics and Statistics Scotland, Edinburgh, UK; ^2^Epidemiology Research Group, Center for Immunity, Infection and Evolution, University of Edinburgh, Edinburgh, UK; ^3^The Roslin Institute, University of Edinburgh, Easter Bush Campus, Edinburgh, UK

**Keywords:** spatial epidemics, kernel transmission functions, Markov Chain Monte Carlo, risk assessment, latent residuals, deviance information criterion

## Abstract

Livestock epidemics have the potential to give rise to significant economic, welfare, and social costs. Incursions of emerging and re-emerging pathogens may lead to small and repeated outbreaks. Analysis of the resulting data is statistically challenging but can inform disease preparedness reducing potential future losses. We present a framework for spatial risk assessment of disease incursions based on data from small localized historic outbreaks. We focus on between-farm spread of livestock pathogens and illustrate our methods by application to data on the small outbreak of Classical Swine Fever (CSF) that occurred in 2000 in East Anglia, UK. We apply models based on continuous time semi-Markov processes, using data-augmentation Markov Chain Monte Carlo techniques within a Bayesian framework to infer disease dynamics and detection from incompletely observed outbreaks. The spatial transmission kernel describing pathogen spread between farms, and the distribution of times between infection and detection, is estimated alongside unobserved exposure times. Our results demonstrate inference is reliable even for relatively small outbreaks when the data-generating model is known. However, associated risk assessments depend strongly on the form of the fitted transmission kernel. Therefore, for real applications, methods are needed to select the most appropriate model in light of the data. We assess standard Deviance Information Criteria (DIC) model selection tools and recently introduced latent residual methods of model assessment, in selecting the functional form of the spatial transmission kernel. These methods are applied to the CSF data, and tested in simulated scenarios which represent field data, but assume the data generation mechanism is known. Analysis of simulated scenarios shows that latent residual methods enable reliable selection of the transmission kernel even for small outbreaks whereas the DIC is less reliable. Moreover, compared with DIC, model choice based on latent residual assessment correlated better with predicted risk.

## 1 Introduction

The livestock epidemics of foot-and-mouth disease (FMD) in the United Kingdom (UK) in 2001 (1) and of classical swine fever (CSF) in the Netherlands in 1997 (2, 3) were characterized by their widespread spatial extent and significant impact on the agricultural sector. The 2001 FMD outbreak affected all UK livestock farms and is estimated to have a cost total of £8 billion (4) whereas the CSF epidemic in Netherlands totalized a short-term economic impact over £1.1 billion (5).

The desire to mitigate such impacts for future livestock-disease incursions has increased focus on preparedness for emerging and re-emerging pathogens (6, 7) and highlighted the need for quantitative tools to support such efforts (8, 9).

A key step in controlling livestock-disease incursions is to quantitatively assess the risk of localized disease spread from infected to susceptible farms. Control strategies can then make use of such risk assessments, for example they can be used to decide on which farms surrounding confirmed infected premises to impose control measures (10, 11). Quantitative study of historical epidemics has the potential to make such risk assessment faster and more robust, enabling more rapid and reliable response than possible if waiting for sufficient data to accrue during an ongoing epidemic (12). Tools to enable analysis of historic outbreaks thus may provide information critical to control operations prompted by future incursions of emerging or re-emerging diseases. Data on large outbreaks such as those described above have been shown to enable quantification of various epidemiological (13), economic (14, 15), and logistical (16) aspects of disease spread. In turn, these studies have contributed to the design of novel, more cost-efficient, control strategies against future epidemics (17–20). Fortunately large outbreaks, such as those described earlier, are rare and incursions of emerging or re-emerging disease typically lead to small and often repeated localized outbreaks (21). In this paper, we will investigate the quality of inference possible from data on small outbreaks.

Model-based inference can be conceptualized as a two-stage process; estimate the parameters for each of a set of given models and then rank or choose between the different models. Subsequent risk assessment can then be based on the model that best fits the data, or suitably weighted outputs from a set of models.

Over recent years, a number of authors have developed Bayesian inferential tools to enable statistically rigorous parameterization of discrete state continuous time Markov and semi-Markov processes (DCTMPs) from noisy and incomplete observations typical of field data, e.g., disease detections from real world epidemics (22–27). These inference tools are flexible in that they can be applied to a wide range of model structures and epidemic scenarios. Discrete state continuous time Markov and semi-Markov processes are well suited to modeling a diverse range of epidemiological systems. Their continuous time nature enables more accurate representation of the transmission process than discrete time models, and their discrete state space is ideally suited to represent epidemic spread between individuals, e.g., farms classified into distinct disease classes, susceptible, infected, etc. The inference tools for such models make use of data-augmentation approaches to account for missing information (such as the times individuals become infected) which are treated as additional parameters to be inferred. Information (including uncertainty) on key quantities obtained from Bayesian inference is encoded in the so-called posterior, which is the joint distribution of model parameters (describing processes such as transmission between hosts and disease progression within an infected host) and missing data, e.g., the infection and transition times conditional on the observed data and modeling assumptions. Markov Chain Monte Carlo (MCMC) methods are typically used to draw samples from the posterior, and from these samples it is straightforward to calculate quantities of interest such as the probability, under some defined scenario, that a given farm will become infected at some future date. It is important to note that such predictions reflect the uncertainty inherent in the inference and therefore indirectly the quality of the data. It can be shown that reliability of inference for a given process (e.g., transmission) increases with the number of associated events (e.g., infections) occurring during the observation period. In general, this supports the intuition that uncertainty in inference will depend on the quantity of data available, and that uncertainty in inferences based on small outbreaks will be greater, and the reliability of the estimates more limited, than for large outbreaks.

Although theory underlying model choice in a Bayesian framework is well established (28, 29), its implementation is often impractical, especially for missing data problems where, as of interest here, latent variables are used for data augmentation. Reversible jump MCMC (30) in principle allows calculation of the so-called Bayes factor comparing two models but suffers implementation issues (31). For example, to compare a newly proposed model with earlier models requires the rerunning (and often recoding) of the inference procedure for at least one earlier model. In practice, the only model selection tool in widespread use for DCTMPs applied to epidemiological modeling is the DIC, or Deviance Information Criteria (32–34). DIC is a Bayesian model selection method which tries to balance model complexity with fit to data (35). However, there are increasing concerns with regards to discriminatory performance (36) particularly in the presence of latent variables, such as infection times, where there is no unique definition of DIC (37). A complimentary approach to model choice is that of model assessment where statistics are constructed to detect inconsistencies between model assumptions and the real world processes being modeled (38, 39). Novel diagnostics tools for assessing the fit of DCTMP model, known as latent residuals, have recently been developed by Lau et al. (40) and have proven to be efficient in identifying misspecification of model components in the context of spatio-temporal models for disease spread. This approach allows indirect model comparison, but also has the important benefit of indicating in what manner a given model may be inadequate, thereby providing insights into ways for improvements. The application of latent residuals for DCTMPs to small outbreaks has yet to be tested (40), and model comparison based on data from small outbreaks is likely to be challenging given the potential difficulties described above associated with inference for individual models from such data.

In this paper, we focus on the spatio-temporal modeling of disease spread between farms and subsequent detection of infected premises, conducting inference from data on detection times only. We focus on the model choice problem of selecting the appropriate functional form describing between-farm disease transmission. Local between-farm transmission is generally mediated by multiple processes influenced by a wide range of factors including human behavior and characteristics of the livestock-disease system in question. However, typically these factors are not quantified and overall local spatial spread is often summarized to be a function of the straight-line distance between farms, modeled by the so-called “kernel transmission function.” In the context of CSF, Mintiens et al. (41) examined risk factors associated with the occurrence of neighborhood infections during the 1994 Belgium outbreak and found that intensity of neighboring herds was the most significant factor. Staubach et al. (42) established a distance-dependent risk function based on data obtained from real outbreaks. Building on this earlier work, Backer et al. (20) modeled between-farm spread of CSF using the kernel transmission function (described later as *K*_2_, see Section 2.1) but modulated the kernel by the infectiousness of the infected farms (infectiousness defined by the number of infectious individuals on farm). The same transmission kernel was considered by Boender et al. (43), but they also included the influence of farm size in order to determine both the distance dependence and the farm-size dependence of the between-farm transmission risk of CSF during the Dutch 1997/1998 epidemic. As we subsequently show, identifying the correct kernel is critical in effective support of policy and decision-making for disease control since it can affect the risk profile of farms that are candidates for control.

In summary, this manuscript is organized as follows:

1. We first focus on the methodology describing the set of models that are mainly different in the kernel transmission functions used. These models’ parameters are inferred in the Bayesian framework and two model selection tools are applied, namely DIC and latent residuals.
2. Having inferred parameters from the various models, we quantify the posterior risks associated with each premise by simulating repeated epidemics using posterior estimates as initial conditions.
3. The reliability of the methods are shown using simulated data and applied to field data on the small CSF outbreak that occurred in 2000 in East Anglia, UK (44).

## 2 Materials and Methods

### 2.1 Model Structure

We use a stochastic spatio-temporal Susceptible-Infectious-Removed (SIR) epidemic model to assess local between-farm spread and farm-level detection and control of a disease. The underlying model framework is a continuous time discrete state-space semi-Markov process. In our model, individuals represent spatially distinct locations, e.g., farms, characterized by a discrete set of disease states (S, I, and R). The population is assumed initially fully susceptible prior to an initial incursion of disease into a single farm (the index case). Susceptible farms (in state S) subsequently become infected via local contacts with infected farms (in state I). Once infected, each farm remains in state I until its infectious status is detected and controls imposed. It is assumed that controls are introduced immediately upon detection and are completely effective. Therefore, once detected farms enter a restricted or controlled state (state R) in which they are removed from (i.e., play no further role in) the epidemic.

#### 2.1.1 Detection and Control

The description of the R state assumes that effective control is rapid following detection which is a reasonable assumption for a range of disease systems (9, 45). In the context of FMD or CSF outbreaks in the UK, total depopulation of the infected premises is carried out within 24 h of the reporting of on-farm detection (1, 44). The infectious period for an infected farm can therefore be reasonably approximate as the period between the date of infection and the date at which the farm in question is reported as infected. The detection of disease is considered non-Markovian since the probability of detection is not constant but varies as a function of time since infection, e.g., as the number of on-farm cases changes. Here, we assume that the infectious period of the disease follows a gamma distribution with shape *α* and rate *γ*.

#### 2.1.2 Secondary Infection

In the situation where farm *i* is infected and infectious (i.e., in state I) and farm *j* is susceptible (i.e., in state S), the rate at which *j* enters state I (i.e., becomes infected/infective) due to transmission from farm *i*, is *β_ij_* = *β*_0_*h_ij_*. Here, *β*_0_ is the maximum rate of contact whereas *h_ij_* is a kernel function that can involve individual-specific characteristics. In the case of a distance kernel function, the contact rate varies as a function of the Euclidean (straight line) distance between farms. The shape of the kernel can significantly impact the spatial spread and development of epidemic processes (46, 47). In order to evaluate the effect of the kernel selection on outbreak risk assessment, we considered four different forms for the kernel function *h_ij_*:

$$\begin{array}{l} K_{1}=\text{exp}\left\{-\tau \rho (i,j)\right\},\\ K_{2}=\frac{1}{1+{\left(\frac{\rho (i,j)}{d}\right)}^{\tau }},\\ K_{3}=\frac{1}{1+\frac{\rho (i,j)}{d}},\\ K_{4}=1-\text{exp}\left(-{\left(\frac{\rho (i,j)}{d}\right)}^{-\tau }\right), \end{array}$$

where *ρ*(*i, j*) denotes the Euclidean distance between individuals or sites *i* and *j* (*i, j* ∈ {1, 2, … *N*}). These functions represent a broad range of kernel functions found in the literature to characterize local spread in the analysis of spatial epidemics. For example, the exponential kernel *K*_1_ was used for modeling the spread of the *citrus tristera* virus (48) and the spread of citrus greening (49). The kernel *K*_2_ was used to model the between-farm spread of the CSF in the Netherlands by Backer et al. (20) and Boender et al. (43). The kernels *K*_2_ and *K*_4_ were used to model the spread of FMD in Japan (50) and in Netherlands (51) and are parameterized in such a way they capture short-range spread. The Cauchy kernel *K*_3_ is a special case of *K*_2_ for *τ* = 1 and was considered by Kypraios (52), and as a description of the dispersal of an invasive non-native vascular plant by Lau et al. (40). *K*_3_ allows more for long-range infections compared to the others as it presents the heaviest tail among all four. Full model specification as given in Section S1.1 in Supplementary Material.

### 2.2 Bayesian Inference

Here, we assume that available data consist of the time at which individual premises were detected as infected, and the geolocations not only of these premises, but all susceptible farms. In this analysis, we assume the times at which individuals are infected are not observed, since this information is typically not available in outbreak situations. However, such information could be incorporated in our framework if it were available.

Missing infection times make the likelihood for the SIR model intractable and render the use of frequentist approaches for inference less than straightforward. However, developments in data-augmentation MCMC allow Bayesian inference of both the parameters and missing infection times. The approach adopted is similar to that of Jewell et al. (25) which builds on the seminal work of Gibson and Renshaw (22) and O’Neill and Roberts (23). Full details on the model fitting framework and procedures are provided in Sections S1.2 and S1.3 in Supplementary Material.

Briefly, we considered the joint posterior distribution of the model parameters ***θ*** and infection times ***I*** given the data, i.e., the detection times ***R***. Using Bayes’ theorem, this can be expressed as

(1) π(θ,I|R)∝π(R,I|θ)π(θ),

where *π*(***R***, ***I***|***θ***) is nothing more than the probability of obtaining a given joint realization of detection and infection times (***I***, ***R***) from a simulation of the model given parameter values ***θ***. Sometimes referred to as the complete data likelihood, this can be calculated based only on the definition of the model. *π*(***θ***) is the joint prior distribution of the parameters and is specified in light of any information available before the data ***R*** is observed. If samples {(***θ****_k_*, ***I****_k_*): *k* = 1 … *L*} can be drawn from the posterior distribution expressed in equation (1), then it is possible to calculate almost any statistic of interest. For example, marginalizing over ***I*** would result in estimating the distribution ***θ****|****R***, whereas marginalizing over ***θ*** would result in estimating ***I***|***R***. We note that the latent space, here corresponding to the set of infection times only, can be readily expanded to consider information such as the transmission tree, i.e., “who-infects-who.” Moreover, the framework can also be expanded to consider other disease categories such as an exposed state.

Here, we employ Markov Chain Monte Carlo (MCMC) methods to draw samples from the posterior distribution specified in equation (1) only up to constant of proportionality. More specifically, we make use of a Metropolis–Hastings (53) within Gibbs algorithm (54) together with simple non-centering techniques (27). Full details of the sampling algorithms are provided in Section S1.3 in Supplementary Material.

### 2.3 Model Selection

Model selection is a challenging aspect of inference, especially for models with latent variables and where data are limited. In this study, we employ two contrasting model selection tools from the literature to select among our four spatial kernels, *K*_1_, …, *K*_4_, which model fits best our data. In particular, we use the deviance information criterion (DIC (35)) and Bayesian latent residuals (40).

Although widely used and measuring the trade-off between the model fit and complexity, DIC is recognized to have issues such as non-invariance to reparameterization, lack of consistency, no basis on a proper predictive criterion (36), and multiple definitions in the presence of latent variables (37). In this study, we used two formulations of DIC for latent variable models (37): DIC_1_ is computed accounting for the full likelihood, i.e., the data augmented likelihood, whereas DIC_2_ is computed on a partial likelihood of the observation process conditional on the latent variables. We note that DIC_1_ and DIC_2_ correspond respectively to DIC_4_ and DIC_8_ in Celeux et al. (37). In either case, interpretation of DIC is straightforward with the smallest value taken to indicate which model performs the best. In practice, however, only differences of magnitude 10 or more between models are usually considered as indicative of significant differences in model fit (55).

By contrast, Bayesian latent residuals based on non-centered reparameterisations of discrete state-space continuous time semi-Markov process have recently been proposed as an approach to assess the fit of different components of the model, thereby focusing attention on aspects of the model that need improvement (40). For example, the infectious link residuals (ILRs) considered here are specifically designed to detect misspecification of the spatial transmission kernel. These are constructed by expanding the set of latent variables sampled from the posterior distribution to include both infection times and also the donors of infection, i.e., the transmission tree. This information then allows inference of a random variable, an ILR, that, under the assumption the model is correct has a uniform *U*(0, 1) distribution (40). Evidence of non-uniform distribution of the ILRs indicates a misspecification of the kernel function. Recall that the data consist only of detection times and the infection times are unknown. Each set of infection times estimated within the MCMC algorithm (i.e., drawn from the posterior distribution) corresponds to a set of residuals. Each of these sets of residuals is subjected to an Anderson–Darling test to assess whether it conforms to a uniform *U*(0, 1) distribution or not. Therefore, there are exactly the same numbers of p-values as there are samples from the posterior distribution. We record the proportion of p-values, e.g., Pr(*p* < 5%), that are less than a confidence level, e.g., 5%. In the case that the proportion is similar to the confidence level, we take that as indicative that the model is not inconsistent with the data; more formally there is not sufficient evidence to reject the model. A high proportion of p-values greater than the 5% level (i.e., Pr(*p* < 5%) large) is interpreted as evidence against the chosen form of the kernel.

To evaluate the performance of these model selection and assessment tools, we apply them within a simulation study framework where the true data-generating model is known. See Section 2.5 for full details of the simulated scenarios considered. For each iteration of the simulation study, we simulate an epidemic and generate a data set (detection times only) using the Gillespie algorithm (56) under a specified kernel. Each dataset is fitted to the models defined in Section 2.1 using MCMC, not only with the kernel used to simulate the epidemic, but also with alternative kernels. Model selection and assessment tools based on DIC and latent residuals are used to assess each of the fitted models as described above. Full details of the model selection tools used are provided in Section S1.4 in Supplementary Material.

### 2.4 Risk Quantification

In order to make the results of inference more directly relevant to the development of disease control, we construct posterior predictive distributions that quantify farm-level risk. To do so, we start by drawing a large number of independent samples from the joint posterior distribution for each model variant (i.e., each kernel). For each kernel, and a fixed set of initial conditions, these sampled parameter values are used to simulate multiple realizations of an epidemic using the Gillespie algorithm from which we record the infected sites and event times. From the large number of simulated epidemic realizations obtained, distributions are calculated for each kernel by evaluating the proportion of realizations in which each premise becomes infected at any given point in time. Such posterior predictive distributions can be used to produce heat maps showing the spatial distribution of farm-level infection risk (see further in section: Results) at a given point in time. The heat maps are produced at a length of times sufficient to capture the early phase and small scale epidemics’ behavior.

An alternative approach to visualizing this information is to summarize the simulated epidemics by looking at the average proportion of infected farms (farm-level prevalence) as function of time. However, interpreting such average between-farm prevalences is complicated by the fundamental characteristics of disease transmission in SIR epidemics. In particular, it is well known that when the basic reproduction rate *R*_0_ > 1 disease spreads on average, but that the final size distribution for a stochastic SIR epidemic model is bimodal, showing that some epidemics die-out before becoming large, while others grow to affect a large proportion of the population (8, 57) (see Section S2.1.3 in Supplementary Material). Given that the mean of a bimodal distribution is not a meaningful summary statistic, we use the mean of the final size distribution as a boundary defining small and large epidemics. This enables us to calculate the probability of obtaining small and large outbreaks and plot average between-farm prevalence as a function of time for both small and large outbreaks, as predicted under each of the four kernels.

### 2.5 Simulation Studies

To assess the reliability of parameter inference and model selection and to evaluate the effect of final epidemic size, we generate data sets based on the Gillespie algorithm (56), introducing the disease via a single randomly selected primary (index) case located in a closed population of *N* = 201 farms in a square of sides [0, 2,000] km (corresponding to an average density of 5 × 10^−5^ farms per 10^2^ km^2^) and using a fixed set of parameters for each scenario considered. The simulation studies described below are divided into simulation study 1 which focuses on single data sets generated using two contrasting kernels, and study 2 which explores coverage properties of our inference procedure using data sets generated from multiple realizations of each of these scenarios. Bayesian inference is applied to every simulated data set to fit models based on all four kernels (*K*_1_, *K*_2_, *K*_3_, *K*_4_), and the model selection procedures described above are applied to every data set to discriminate between the kernels.

#### 2.5.1 Simulation Study 1a

We first simulate a single realization of the epidemic assuming that the mechanism of disease spread is described by kernel *K*_1_. More specifically the characteristics of underlying infection process are *β*_0_ = 0.35, *K*_1_ = exp{−0.008*ρ*(*i,j*)} with an initial condition in which all farms are susceptible except for a single, randomly selected primary case. The infectious period follows a Ga(5, 5) distribution. A total of *n_R_* = 43 removed individuals were recorded together with their removal times.

#### 2.5.2 Simulation Study 1b

A single data set of detection times is generated from a simulation using kernel *K*_2_ instead of *K*_1_. The detection time distribution and initial conditions are as in study 1a but here, *β*_0_ = 400, $K_{2}=\frac{1}{1+{\left(\frac{\rho (i,j)}{1.5}\right)}^{2}}$, and *n_R_* = 44 removal times were obtained.

#### 2.5.3 Simulation Study 2a

Different outbreak sizes are considered in order to assess goodness-of-fit and evaluate the performance of model selection tools as epidemic size increases. Simulations are performed to obtain *n* = 30 realizations for each epidemic size category of [6, 10], [11, 15], [16, 20], [21, 25], [26, 30], [31, 35], [36, 40], and [41, 45]. For each realization, a data set of detection times was recorded. For each randomly selected incursion event, the spread of the disease was simulated using kernel transmission function *K*_1_ with the same parameterization used in simulation study 1a. Considering only small (≤45 infected farms) completed epidemics, we subsequently inferred all parameters of interests (as well as posterior distributions of infection times for infected premises), and computed all three measures of goodness-of-fit (DIC_1_, DIC_2_, ILR) under the hypothesis that the spread of the disease follows a kernel transmission function with a shape either *K*_1_, *K*_2_, *K*_3_, or *K*_4_. For each scenario, coverage properties (i.e., the number of times the true parameter values fall within their respective 95% credible intervals) are recorded for each size category described above.

#### 2.5.4 Simulation Study 2b

To evaluate the resilience of our conclusions given to the shape of the kernel transmission function of the data generation process, an analogous procedure to that described for study 2a was carried out considering data generated using *K*_2_ (i.e., instead of the kernel transmission function *K*_1_) with the same parameterization as used in simulation study 1b.

### 2.6 Field Data

In 2000, the UK experienced an outbreak of CSF across the region of East Anglia (44). Unlike in the Netherlands where the CSF outbreak has been detected in various areas of the country (58), the UK outbreak was only detected in the region of East Anglia, with n = 16 farms found infected in the 3 month-long outbreak. Records of the pig population at the time show that N = 1,703 pig farms were present in the affected area and were considered at risk (44). This represents an approximate average population density of 6.08 per 10^2^ km^2^. Although all pig farms in the UK could be considered at risk, inferred transmission distances are small relative to the area occupied by the above subpopulation, and all infected premises are contained comfortably in the defined region and the risks for farms outside the subpopulation considered are considered negligible given their distances to the infected farms and the inferred transmission kernels (see Results section), unless sources of infection exist other than those considered in the modeling framework used here. The inferences presented below would therefore be essentially unchanged by accounting for a larger at risk population.

All parameters of interest including infection times were inferred using data describing farm locations and the time at which each premises was detected and reported infected. Control interventions consisted of depopulation of infected premises within 24 h of reporting of disease detection. Inference was conducted, and all model selection criteria and measures of goodness-of-fit computed, under the hypothesis that spread of the disease was described by the spatial SIR model described above with each of the kernel transmission functions *K*_1_, *K*_2_, *K*_3_, or *K*_4_. Although CSF is highly contagious and may result in the death of young animals, clinical sign are non-specific and can result in failed diagnosis and a long period of undiagnosed spread (59). Consistent with the literature (20), we therefore assumed that a minimum of 8 days was required for infected premises to be detected. Therefore, during the fitting procedure, the infectious period was assumed to follow a left-truncated gamma distribution. Full details are available in Section S1.1 in Supplementary Material.

## 3 Results

### 3.1 Testing Inference Methods with Simulated Data

We first make use of simulated data to assess the performance of inference and model selection procedures and risk assessments based on them. Initially the focus is on inference from individual epidemics, but latterly we explore coverage properties, as a function of outbreak size, by considering performance on data from a representative ensemble of epidemics.

#### 3.1.1 Inference from Single Epidemics

Posterior means for model *K*_1_ parameters, under simulation study 1a, were 0.396 (95% credible interval 0.169, 0.761), 6.251 (2.638, 11.631), 0.00771 (0.00540, 0.01031), and 4.907 (1.986, 9.190), respectively, for *β*_0_, *γ, τ*, and *α*. All 95% credible intervals overlap with the underlying parameters values used to generate the data, suggesting our inference framework is able to recover appropriate parameterizations when there is no mismatch between the data-generating mechanism and fitted model. This is also the case for simulation study 1b which replaces kernel *K*_1_ with *K*_2_ and inference for *β*_0_, *γ, τ, α*, and *d* yields estimates 714.576 (59.319, 2,809.770), 7.931 (3.298, 13.429), 2.016 (1.639, 2.411), 5.567 (2.228, 9.782), and 2.237 (0.699, 4.717), respectively. Assessments of convergence of the outputs of the MCMC sampler reveal no evidence of lack of convergence as shown by the auto-correlation functions in Section S2.1.1 in Supplementary Material.

Table 1 shows the values obtained for each of the model selection tools implemented when assessing the fit of model variants based on each of the four transmission kernels. The correct kernel was identified when considering the ILRs making use of the measure Pr(*p* < 5%), the proportion of p-values less than 5% based on the latent residuals, and using DIC_1_. It is also worth noting that analysis of the residuals suggests an equally good fit for *K*_2_ and *K*_4_ in simulation study 1b. By contrast, DIC_2_ led to selection of the incorrect kernel in both situations, preferring *K*_3_ rather than either *K*_1_ or *K*_2_. This finding is consistent with previous studies (40) and highlights that using DIC_2_ may generate selection bias.

**Table 1:** **Computed DIC_1_ and DIC_2_ values and the proportion of p-values less than 5% obtained from testing the distribution of ILRs when fitting model variants *K*_1_, …, *K*_4_ to simulated data from scenarios 1a (*a*) and 1b (*b*)**.

(*a*) Simulation study 1a: true kernel, *K*_1_			

	DIC_1_	DIC_2_	Pr(*p* < 5%)
*K*_1_	**266**	761	**6.42%**
*K*_2_	274	23,741	36.88%
*K*_3_	285	**630**	90.99%
*K*_4_	273	39,486	39.29%

**(*b*) Simulation study 1b: true kernel, *K*_2_**			

	**DIC_1_**	**DIC_2_**	**Pr(*p* < 5%)**

*K*_1_	239	371	34.79%
*K*_2_	**227**	494	**4.43%**
*K*_3_	252	**362**	89.61%
*K*_4_	394	489	4.64%

*Bold font indicates the smallest values for the DICs and Pr(*p* < 5%) bringing out the selected model by each tool*.

To evaluate the implication of such miss-selection on predicted risk associated with different premises, we construct posterior predictive probabilities of infection under all kernels (corresponding risk maps are shown in Section S2.1.4 in Supplementary Material). Comparing across kernels there are clear differences in risk levels predicted for many locations. *K*_3_ predicts highest risk of infection for all farms while predicted risk under *K*_2_ and *K*_4_ decreases for extreme locations, whereas the predicted risk under *K*_1_ falls off much faster with distance from the index case.

**Figure 1:**
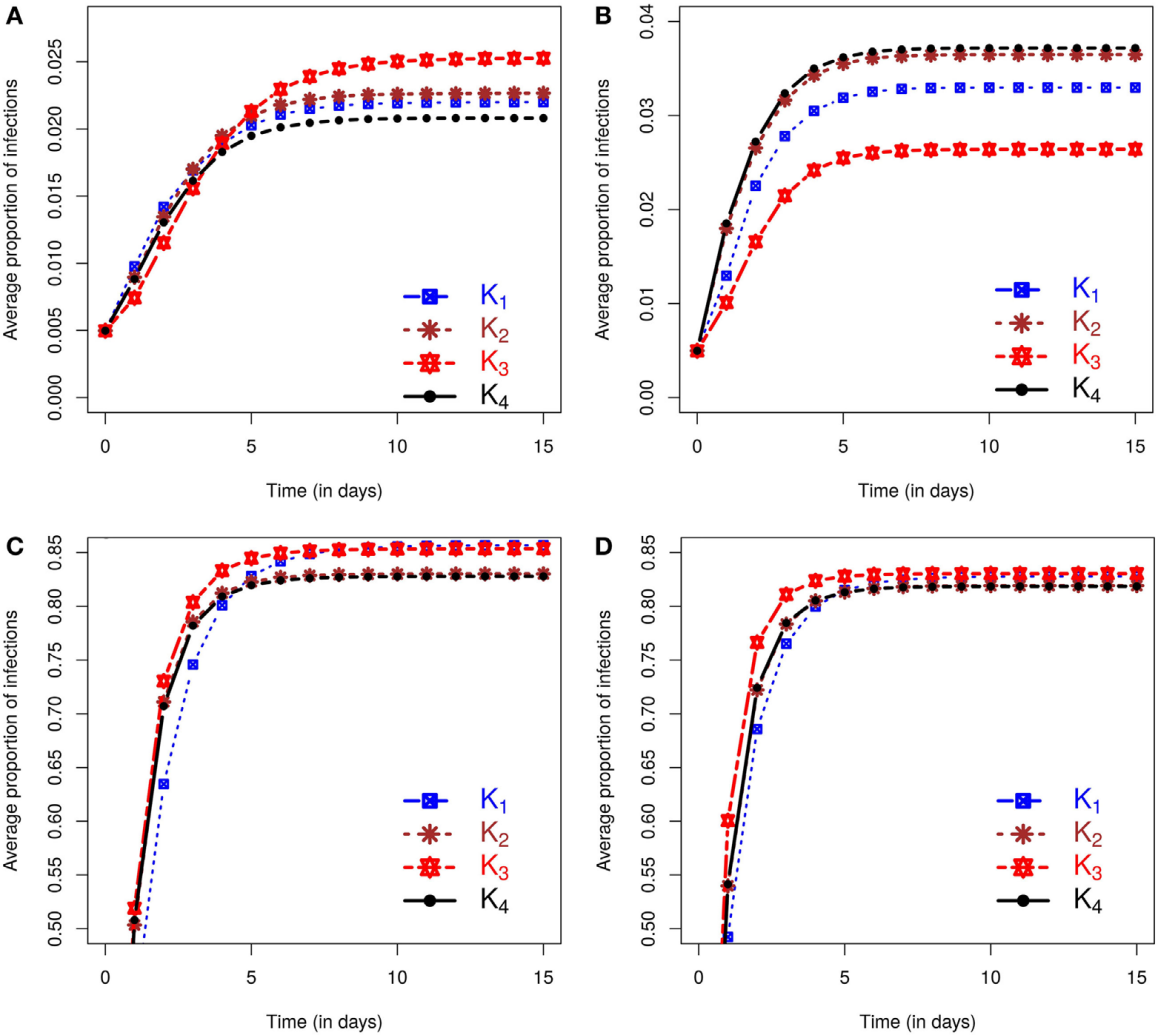
**Posterior predicted average proportion of premises infected as the epidemics evolve in time (days)**. On each graph, the lines correspond to the results obtained when kernels *K*_1_ − *K*_4_ are fitted to data. The column on the left shows results using data from simulation study 1a and is divided into predicted outbreaks that are **(A)** small or **(C)** large. The column on the right shows results from simulation study 1b, stratified for **(B)** small or **(D)** large predicted outbreaks. The size of outbreaks was classified as either small or large based on final outbreak sizes being smaller or larger than the mean of the final size distribution (see text for details).

Figure 1 shows separately for both small and large outbreaks, how the average proportion of infections, i.e., the total number of cases divided the total number of farms within the area, evolves through time under different kernels. Small (large) outbreaks are defined as those less (greater) than the posterior mean epidemic size (see methods). The between kernel differences in the posterior predicted average proportion of infected premises through time, are very noticeable for small epidemics, and still visible, but to a lesser extent, for large epidemics.

We also evaluated the posterior predicted risk of a small as opposed to a large outbreak. Under simulation study 1a small epidemics occur 61.87% of times for *K*_1_, 58.59% for *K*_2_, and 52.88% and 59.12% for *K*_3_ and *K*_4_, respectively. Similar proportions are obtained under simulation study 1b except for small difference in *K*_3_ with 57.18%, while other proportions are 60.39%, 59.03%, and 58.83% for *K*_1_, *K*_2_, and *K*_4_, respectively. It is also worth noting that in both simulation studies 1a and 1b, the observed epidemics on which inference is based are classed as small, but these lie at the upper end of the final size distribution for small epidemics according to the outbreak size classification used here. Inference from these single outbreaks therefore captures the true underlying bimodal nature of the outbreak risk associated with the SIR dynamic (see Section S2.1.3 in Supplementary Material). In terms of assessment of risks associated with future incursions this means that the probability of obtaining an outbreak similar in size to the observed outbreak is relatively low.

The posterior predicted estimates of future outbreaks produce rather different risk assessments depending on the kernel used to fit the data, suggesting that reliable model choice is of key importance.

In assessing the model selection criteria studied here several key points are noteworthy. First, the ability of DIC_2_ to identify the correct model is poor, whereas DIC_1_ and the ILRs do so in both simulation study 1a and 1b. Second, different kernels lead to different predicted risk. For example, the model variant with kernel *K*_3_ produced the most extreme predictions of risk. However, this was also the model least favored when testing model assumptions using the ILRs, but was not consistently ranked by DIC_1_. In simulation study 1b, analysis of ILRs leads to selection of two models, i.e., with kernels *K*_2_ and *K*_4_, and these models produced almost identical posterior predicted risk profiles in terms of expected proportion of infected farms. This similarity is not evident in their DIC_1_ scores. These results suggest that model selection based on the ILRs may be better able to identify differences in posterior predicted risk profiles than DIC.

#### 3.1.2 Coverage Properties and Effect of Outbreak Size

To generalize our findings, we considered epidemics generated with either a *K*_1_ or *K*_2_ kernel function and evaluated how inferences (description in section *Bayesian Inference*) and model selection tools (details given in section *Model selection*) considered may perform as a function of the epidemic size and across multiple realizations of the epidemic process (see sections *Simulation study 2a* and *Simulation study 2b*). Coverage properties of our inference procedure from multiple realizations are explored and available in Section S2.2 in Supplementary Material. The coverage rates obtained show that the true parameters are contained approximately 95% of the time in their corresponding credible intervals. However, the rates are higher for the parameters of the infectious period distributions where informative priors are used on the shape parameter as in Kypraios (52) and Streftaris and Gibson (60). The uncertainty of the estimates reduces as the epidemic size increases.

**Figure 2:**
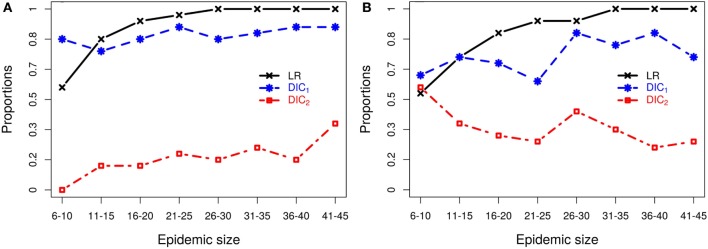
**Probabilities of correctly selecting the right model using latent residuals (LR), DIC_1_, and DIC_2_ in the case of (A) simulation study 2a (using *K*_1_ to simulate the data) and (B) simulation study 2b (using *K*_2_ to simulate the data)**. Both graphs show that the LR perform better (higher probabilities) in selecting the right kernels than the DICs as the epidemic size increases.

For each epidemic size category, Figure 2 plots the proportion of simulated data sets from which each of the model selection criteria successfully identifies the correct kernel. Figure 2 shows that, although inferences of model structure are reasonably accurate for small epidemics, increasing epidemic size increases the accuracy with which the underlying infection process is identified. However, this depends on which measures of goodness-of-fit are used. While the DIC measures contradict each other, the latent residuals typically distinguish between the kernels and select the correct model used to simulate the outbreak (e.g., Figure 2A). However, for the smallest outbreaks, DIC_1_ has the best record (e.g., Figure 2B), but as epidemic size increases, the reliability of the latent residuals quickly improves and outperforms both DIC_1_ and DIC_2_. For epidemic sizes greater than 20, ILRs identify the correct model in at least 90% of cases. In all situations considered, DIC_2_ provided contradictory results to DIC_1_, maintained a low success rate in choosing the true model, which for scenario 2b actually declined with outbreak size.

Figure 2B illustrates the resulting patterns from simulation study 2b which uses models based on *K*_2_ to generate the data. This plot shows results for fitting only *K*_1_–*K*_3_, and *K*_4_ is not considered since as we saw above *K*_2_ and *K*_4_ are difficult to discriminate, but lead to similar risk assessments. Including both here, therefore gives a biased view of the model selection process (see Section S3 in Supplementary Material). To better understand why these kernels are not efficiently discriminated, we explored the effect of the density of the population at risk. Results (details shown in Section S3 in Supplementary Material) show that increasing the density of farms provides more information about short-range transmission (i.e., more short-range transmissions occur) allowing differences between *K*_2_ and *K*_4_ to be distinguished. For realistic range of parameters, *K*_2_ and *K*_4_ can have similar long-range behavior or similar short-range behavior, but not both and therefore can agree when fitted to data which largely excludes short-range transmission, as is the case when the population density is low. These results highlight the critical importance of population density in the local spread of disease. They also explain the effect seen here where we are unable to discriminate between kernels *K*_2_ and *K*_4_ using simulated data from a low density population (5 × 10^−5^ per 10^2^ km^2^), but are able to detect differences between these kernels when fitting to field data below (see section *Classical Swine Fever epidemic*) where the density of farms is considerably higher (6.08 per 10^2^ km^2^).

### 3.2 Classical Swine Fever Epidemic

We now apply the inference and model selection methodologies described above to field data from a small scale outbreak of CSF in East Anglia, UK in 2000.

As described in section *Materials and Methods*, stochastic spatio-temporal models were fitted to the CSF outbreak field data (see section *Field data* for details) using each of the kernels *K*_1_–*K*_4_. This process yielded estimates of a number of quantities that are difficult to measure directly including the unobserved infection times, the infectious period distribution and the transmission kernel. In general, the inferred infection times agree well with the independent estimates obtained through contact tracing procedures during control activities as shown in Section S2.4 in Supplementary Material. The average infection times suggest that infections from first to last infected farm happen in an interval of approximately 65 days and around 9 farms were infected before the first detection.

As expected, the inferred transmission kernel functions all decay as a function of distance but vary according to the fitted form. Posterior medians and their corresponding 95% credible intervals of the kernel transmission function are plotted on a log-scale under the four different kernels, with details on model goodness-of-fit and convergence in Section S2.4 in Supplementary Material. *K*_1_ seems to decay fastest with the widest 95% credible interval. As before *K*_2_ and *K*_4_ look very similar in terms of median shape and credible interval and seem to allow for close range transmission, while *K*_3_ decays very slowly and presents the smallest 95% credible interval. By contrast, choice of the transmission kernel has little impact on the inferred infectious period (Section S2.4 in Supplementary Material).

**Figure 3:**
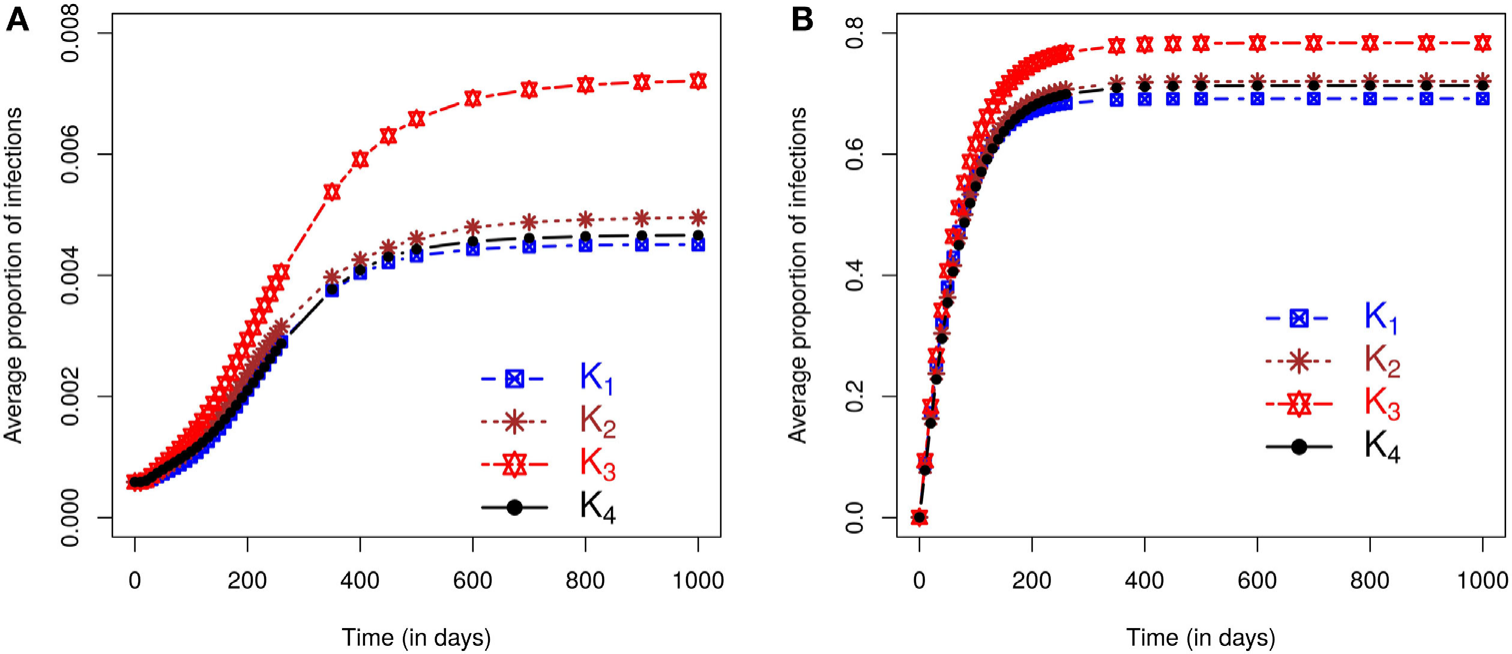
**Posterior predicted average proportion of premises infected plotted as a function of time**. On each graph, the lines correspond to the results obtained when kernels *K*_1_ − *K*_4_ are fitted to the CSF data with final sizes **(A)** smaller or **(B)** larger than the mean of the final size distribution.

We now evaluate the impacts of each kernel on posterior predicted risks. Figure 3 displays the mean posterior predicted proportion of infected farms as a function of time. Predictions of this risk measure under *K*_3_ anticipate a larger number of infected premises, both in the case of small and large outbreaks, when compared with predictions based on the other kernels. The predictions under the remaining kernels are similar, and once again the differences between *K*_2_ and *K*_4_ are particularly small. As before, small (large) outbreaks are defined here as those where the final epidemic size is smaller (larger) than the mean of the final size distribution. The similarity between *K*_2_ and *K*_4_ and the relative difference of predictions under *K*_3_ also hold for the inferred probabilities of a small outbreak, which are 65.45%, 66.17%, 63.75%, and 66.17%, respectively, under kernels *K*_1_, *K*_2_, *K*_3_, and *K*_4_. As with the simulation studies, the observed epidemic was classified as small but with the proportion of infections observed in the real epidemic greater than the average size predicted for small outbreaks (see plot in Section S2.5 in Supplementary Material).

**Figure 4:**
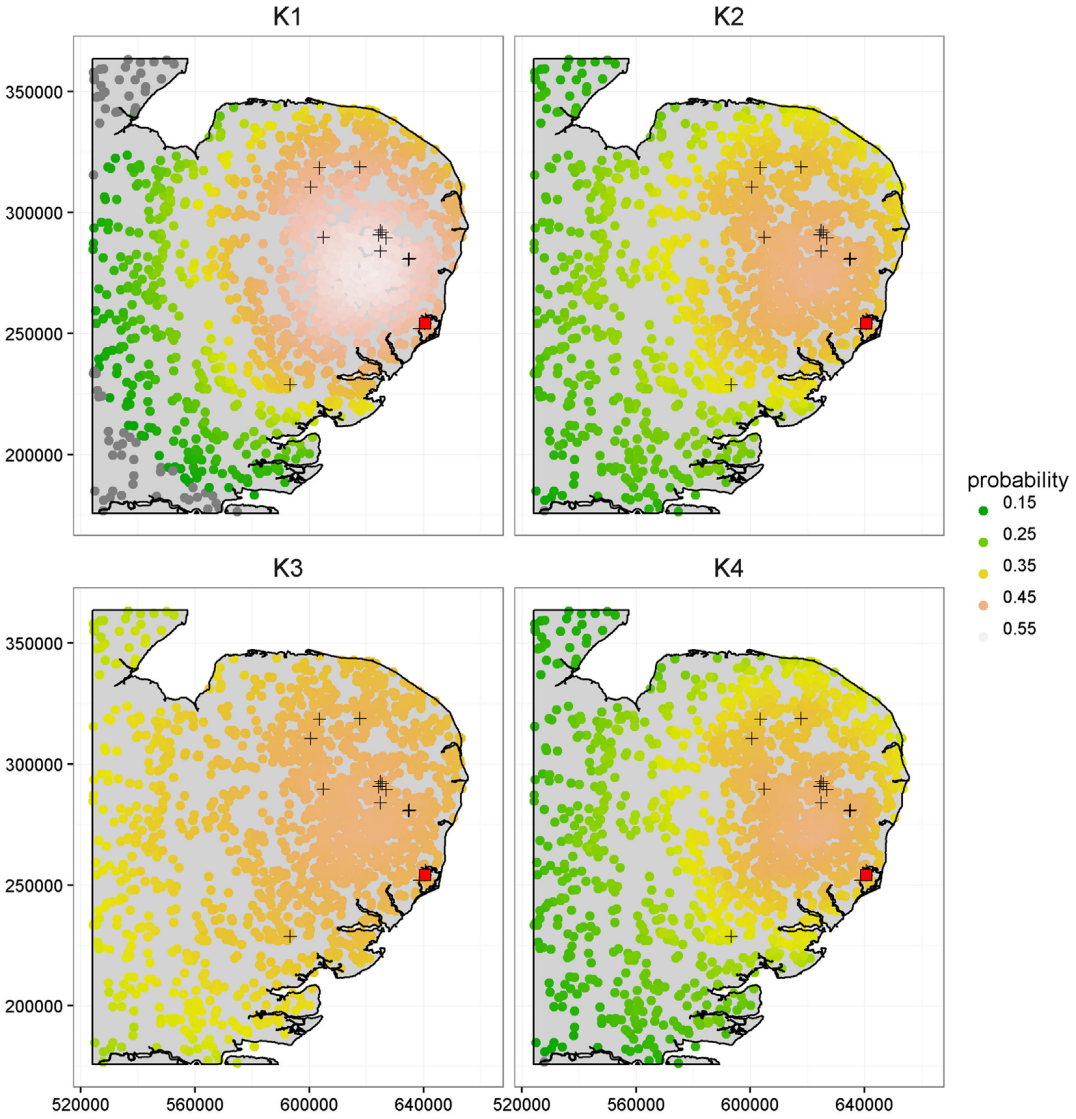
**Comparison of the risk maps using *K*_1_, *K*_2_, *K*_3_, and *K*_4_ at time *t* = 90 days, corresponding to a length of time sufficient to capture the early phase and small scale epidemics’ behavior, based on the population from the CSF data**. The 16 cases detected during the real outbreak are shown by the “+” symbols, along with the index case shown as a red square. The x and y axes are in meters.

Figure 4 further illustrates the importance of kernel choice by plotting risk maps of the probabilities of infection of each farm for a disease incursion. While farms close to the index case seem to present the highest risks of infection across all maps, the risk profiles of farms look different as we get further from the index case under each kernel. Figure 4 shows that predicted risks under *K*_3_ are the highest for farms situated at the largest distances from the index case because of its long right tail. At intermediate distances, the risks of farms under kernel *K*_1_ are actually the greatest, but predicted risk is the lowest and decreases quickly for long distances compared to the other kernels. Kernels *K*_2_ and *K*_4_ predict visually similar risk with a consistent pattern of observed cases in terms of spatial extent and intensity of infection within the high risk area.

It is worth noting that most observed cases during the real CSF outbreak have a relatively high risk profile with an average probability of infection by *t* = 90 days of {0.52, 0.46, 0.46, 0.45} under *K*_1_, *K*_2_, *K*_3_, and *K*_4_, respectively, while the average risk across all farms is {0.41, 0.36, 0.40, 0.35}, i.e., a difference of {0.11, 0.10, 0.06, 0.10}. The different risk predictions under the four kernels are further quantified in Table 2 which shows the number of farms with expected posterior probability of infection by *t* = 90 days less than 0.15, between 0.35 and 0.45, and greater than 0.45. Most farms showed probability of infection between 0.35 and 0.45 for *K*_3_ while predictions under *K*_1_ show more farms in this highest risk category. These figures also reveal relatively subtle differences in predicted risk under *K*_2_ and *K*_4_. The contrasting risk profiles explored above provide a further demonstration of the importance of kernel choice in predicting risk and therefore in terms of the design of disease control programs. Therefore, we now turn to our model selection methods.

**Table 2:** **Summary of model fit and risk assessments based on the CSF data: the results are provided for each kernel *K*_1_ − *K*_4_ as indicated in the first column**.

	DIC_1_	DIC_2_	Pr(*p* < 5%)	*n*(risk > 0.45)	*n*(risk ∈ (0.35, 0.45))	*n*(risk < 0.15)
*K*_1_	429	156	27.78%	866	395	72
*K*_2_	**317**	157	**10.67%**	105	954	1
*K*_3_	353	**156**	32.83%	1	1,466	0
*K*_4_	411	158	19.47%	15	933	1

*The next two columns indicate the computed DIC_1_ and DIC_2_ values with their smallest values under *K*_2_ and *K*_3_, respectively. The following column reports the proportion of p-values less than 5% (Pr(*p* < 5%)), resulting from testing the distribution of ILRs at each MCMC iteration, with the smallest proportion occurring under *K*_2_. The other columns show the number of farms falling in defined intervals of probabilities of infection at time *t* = 90 days under the four kernels*.

*Bold font indicates the smallest values for the DICs and Pr(*p* < 5%) bringing out the selected model by each tool*.

As previously, we used DIC_1_ and DIC_2_ and the latent residual methodology to assess the suitability of the four kernel transmission functions in light of the available data. The latent residuals method gave a preference to *K*_2_, followed in order of choice by *K*_4_, *K*_1_, and *K*_3_, respectively, as shown in Table 2. From a purely model assessment perspective, there is some evidence against all the kernels using the latent residuals since Pr(*p* < 5%) is greater than 5% in all cases, but this is hardly surprising given the relative simplicity of the models used. However, in terms of model selection, *K*_2_ is preferred. DIC_1_ agrees with the latent residuals method in the choice of *K*_2_ as the preferred kernel but the assessments are not in the same order for the other kernels. By contrast, DIC_2_ values do not show significant differences between kernels.

## 4 Discussion

The potential for large scale livestock epidemics to give rise to significant economic, welfare, and social costs (see, e.g., Ref (4, 5)) emphasizes the need for quantitative assessment (8, 9) of the risks associated with emerging and re-emerging pathogens. There is potential to use small localized outbreaks of emerging or re-emerging pathogens to inform such risk assessments before a large outbreak occurs. The key challenge addressed in this paper is to use data from small localized historic outbreaks (21) to inform quantitative risk assessment. We show that rigorous risk assessment based on small outbreaks can be achieved by combining state of the art methods for statistical inference in stochastic epidemic models. Moreover, this methodology was tested using simulated scenarios and by application to data on a small outbreak of CSF in East Anglia, UK (44).

We have shown that data-augmentation MCMC techniques (23, 25, 27) can be applied to continuous time models in order to generate Bayesian estimates of key characteristics of between-farm epidemics using data from small outbreaks consisting of farm locations and times at which disease is detected on farm. These estimated characteristics, impractical or difficult to measure directly, include unobserved exposure times, the distribution of times between exposure and disease detection, and the so-called transmission kernel describing the nature of spatial spread of disease between farms.

Analysis of inferences based on simulated data scenarios shows that when the fitted model has the same form as that used to generate the data, inferred parameter estimates are reliable even when using data from relatively small outbreaks, and the precision of such estimates increases with outbreak size. Fitted models can be used to conduct risk assessments of future outbreak scenarios that account for the uncertainty in parameter estimates. Such predicted risks are said to be drawn from a posterior predictive density and can be used to inform disease control efforts, e.g., targeting high risk farms (18, 61). Therefore, the method has the potential to inform the design and implementation of control measures such as the size of control zones used as part of wider movement restrictions and the geographically targeted use of vaccine and removal operations (17, 50, 62).

However, when analyzing real disease outbreaks all implementable models are, to varying degrees, approximations of the underlying system (63). We therefore considered scenarios in which the data were generated using a known model, and then used as the basis of inference for a set of models which varied according to the functional form of the spatial transmission kernel. Since the resulting risk assessments were found to be dependent on the structure of the model being fitted, model choice has important implications for such disease control policies.

Although risk assessment could be based on a weighted average across the set of models considered, here we focused on criteria used to select a single best-fit model. In particular, we considered two forms of the Deviance Information Criteria (DIC) and model selection based on analysis of so-called latent residuals (see methods for details). DIC is not uniquely defined for inference problems involving latent variables, e.g., missing exposure times, and we considered two variants, DIC_1_ and DIC_2_ (37). Latent residuals are designed to assess the fit of particular model components and we focused on Infectious Link Residuals (ILRs) to test the appropriateness of the form of the kernel density function (40). The set of fitted models included the data-generating model thus enabling an objective assessment of these model selection procedures. Multiple simulated replicate data sets for a range of outbreak sizes were generated and fitted so that coverage properties of inferences could be determined and reliability of model selection procedures assessed as a function of outbreak size.

For the scenarios considered it was found that DIC_2_ was unreliable, but that model selection based on either DIC_1_ or ILRs achieved high levels of reliability even when using data from relatively small outbreaks. For outbreak sizes of 11–15 and above, model selection based on ILRs out performed that based on DIC_1_. For outbreaks with more than 20 cases, there is at least 90*%* of chance of selecting the correct kernel using ILRs, increasing to 100% for outbreaks with 31–35 cases or greater. However, reliability falls off with outbreak size and for data sets containing 6–10 cases, analysis of ILRs identified the data-generating model in a little over 50% of cases. In this very low data regime, DIC_1_ was seen to give slightly more reliable model selection.

Ranking of models based on ILRs was more closely correlated, in comparison with DIC based assessments, with predicted risks under future disease incursion scenarios. For example, we found that two of the four transmission kernels considered in this study provided similar fits to the data especially when the density of farms was low and the number of short-range transmissions limited. In such scenarios, it was found that the kernel *K*_4_ and a generalized form of the Cauchy kernel *K*_2_ that captures short-range spread, predicted very similar risk profiles. Moreover, this similarity was reflected in model rankings based on the infectious link residuals, but not in DIC values. When farm density was higher a greater number of short-range transmissions were inferred and differences between *K*_4_ and *K*_2_ were evident in both rankings based on ILRs and in the posterior predicted risk profiles. In the case of field data on CSF, we found subtle differences in the risk profiles predicted under these two kernels, and this was flagged by analysis of the ILRs. However, the similarity between predictions under these models was not reflected in their DIC scores.

We have illustrated our approach using a particular set of between-farm epidemic models applicable to CSF outbreaks. However, the methodology is flexible and could be applied to a broad range of models, e.g., incorporating additional disease classes, multiple diagnostic tests, or the modeling of specific routes of infection. Here, we considered kernel transmission functions based on Euclidean distance and in many cases, the detailed information needed to parameterize more specific routes of infection may not be available. Euclidean distance-based kernel transmission functions have been extensively used by many authors (48–52) and according to Savill et al. (64), Euclidean distance is better predictor of transmission risk than shortest and quickest routes via road, and appropriate to most regions except where major geographical features intervene.

We tackled the challenging problem of extracting useful information from a knowledge of just the location of the susceptible population of farms and farm-level case detection data obtained from observations of small sized epidemic outbreaks (16 cases for the CSF epidemic). We have shown that using such limited data it is possible to perform reliable inferences and quantify disease risks associated with individual farms. The work reported here focused on SIR epidemic models. In the case of CSF within individual animals, there is evidence of an exposed class, E, suggesting that for an individual level model an SEIR model would be more appropriate (20). However, in this paper the farm was taken to be the basic epidemiological unit since information was only available describing the infectious status of whole farms and following Stegeman et al. (58) farms were categorized according to an SIR framework. Definition of the exposed state at the whole farm level is somewhat problematic since exposed individuals may be moved between farms and therefore spread infection. We note that in modeling between-farm transmission, Boender et al. (43) do not consider an exposed farm state explicitly but do modulate for farm size. An interesting focus of future work could be a formal statistical assessment of SEIR versus SIR models of between-farm spread. However, in the small outbreak setting this would be challenging given the limited information available in the data. Other authors have sought to tackle limitations in observed case data by developing approaches that combine phylogenetic information with the case detections to increase the power of estimates (65–67). However, the methods presented here are applicable in the many situations where suitable phylogenetic data is not available.

The underlying methods described in this paper are generic and could be applied to a wide range of disease scenarios including other livestock diseases. However, while the methodology is generic it is critical to tailor models to the scenario of interest to represent key aspects of the disease dynamics and the available data. For example, such models could account for the role of wildlife in disease transmission, but in practice how this could be achieved is dependent on the extent to which data is available on the prevalence of the focal pathogen in the wildlife host (or hosts). In the absence of such data, it is likely that at best it may be possible to estimate a background infection rate from wildlife sources. Another interesting possibility would be to simultaneously model the spread of multiple pathogens including interactions between them, but again this would only yield meaningful estimates if suitable data were available.

In conclusion, we have developed a toolkit to reliably assess risks from potential future disease incursions using observational data from historic outbreaks that can be applied to support policy decisions relevant to contingency planning for emerging and re-emerging pathogens. We have shown that epidemic models based on discrete state continuous time Markov and semi-Markov processes and data-augmentation MCMC techniques enable reliable and rigorous statistical inferences and probabilistic risk assessments based on data from relatively small between-farm outbreaks. Moreover, recently introduced model assessment methodology based on latent residuals (40) enables candidate models to be ranked, on the basis of their fit to the available data, in a manner that is more reliable than standard DIC approaches (35, 37). We tested this toolkit in the data limited regime using both simulated data and by application to a real world outbreak of CSF with only 16 infected farms.

Our approach is designed to make the best possible use of the data available from even very small historic outbreaks. However, it is important to realize that such data may provide a biased view of future incursions. For example, if the region in which the historic outbreak occurred is not representative of the regions for which risk assessments are needed, then estimates obtained, e.g., of rates of transmission need to be applied with suitable caution. Nonetheless, our approach does provide a rational and statistically rigorous approach to extracting information on disease dynamics and transmission characteristics that are difficult, costly or impossible to measure directly. The quantification of such characteristics and associated uncertainty provides a practical and rational basis for the quantitative assessment of risks under future pathogen incursions.

## Ethics Statement

Ethical approval not required as the data were collected from clinical examinations arising from a natural outbreak.

## Author Contributions

KG, GM, and TP conceptualized the ideas and formulated the goals. KG and GM developed and designed the methodology with the formal analyses carried by KG. TP provided the outbreak data and all authors contributed to the writing of the manuscript. GM secured funding for this project.

## Conflict of Interest Statement

The authors declare that the research was conducted in the absence of any commercial or financial relationships that could be construed as a potential conflict of interest.
