# Supplementary material for: Data-Driven Risk Assessment from Small Scale Epidemics: Estimation and Model Choice for Spatio-Temporal Data with Application to a Classical Swine Fever Outbreak
Source: Front Vet Sci. 2017 Feb 28;4:16. doi: 10.3389/fvets.2017.00016 (PMC5329025; doi:10.3389/fvets.2017.00016)
Supplement: Supplementary file 1 [file presentation_1.pdf]

Supplementary Material: Data Driven Risk Assessment from Small Scale Epidemics: Estimation and Model Choice for Spatio-Temporal Data with application to Classical Swine Fever Outbreak

Kokouvi Gamado, Glenn Marion* and Thibaud Porphyre
*Correspondence:
Glenn Marion
Biomathematics and Statistics Scotland
JCMB, The King's Buildings
Peter Guthrie Tait Road
Edinburgh, EH9 3FD
Scotland, UK
Glenn.Marion@bioss.ac.uk

1 METHOD DESCRIPTION

1.1 Model framework

The stochastic SIR epidemic model where individuals go through the stage of susceptible to become infectious and then removed or recovered can be used to reflect the different status of farms during an outbreak (Dawson et al., 2015). The infectious compartment corresponds to the status where the farm is infected and is able to transmit the disease to other sites; while the recovery state means that the infection has been detected in the farm and such farm has been isolated or restrictions of activities that would affect others are in place. Other formulations of compartment models for between farm epidemics consider stages of exposure where the farm go through a latent period before it can transmit disease (Streftaris and Gibson, 2012; Keeling and Rohani, 2007) or notification as the farm is known infectious but the control measures limit its impact on the network (Jewell et al., 2009). In the model below, we assume that there is no movement between farms or the movement is reflected in their distance apart.

In a closed population of N individuals where each individual's exact position in the space is known, we assume that an epidemic starts with a single initially infected individual.

The susceptible to infectious part of the process as follows:
An individual i makes an infectious contact with a susceptible individual j at rate β_{ij} which we assume to be

$$\beta_{ij} = \beta_0 h_{ij}$$

where β_0 is the contact rate. The expression h_{ij} can take various forms depending on the epidemic studied and the belief of how the disease spreads. In the present paper, the study will focus on four shape of h_{ij} which together with β_0 represent well known spatial kernel transmission functions:

1. K_1 (usually known as the exponential kernel)

$$h_{ij} = \exp\{-\tau\rho(i,j)\} \tag{S1}$$

2. K_2

$$h_{ij} = \frac{1}{1+\left(\frac{\rho(i,j)}{d}\right)^{\tau}} \tag{S2}$$

3. K_3 (the so-called Cauchy kernel)

$$h_{ij} = \frac{1}{1+\frac{\rho(i,j)}{d}} \tag{S3}$$

which is a special case of the Kernel 2 with $\tau = 1$

4. K_4

$$h_{ij} = 1-\exp\left(-\left(\frac{\rho(i,j)}{d}\right)^{-\tau}\right) \tag{S4}$$

where $\rho(i,j)$ denotes the Euclidean distance between individuals or sites i and j $(i,j \in \{1,2,...N\})$. The distance kernels as defined above allow the infection rates to decrease when the distance between two individuals decreases.

The process from infectious to removal is modelled as follows:
For the real epidemic data we consider in the model that removal or detection of diseases in a farm are through tests and the tests only happen at fixed dates. As a consequence, an infected/infectious individual becomes removed/detected after a minimum of c days of being infectious. The infectious period of the epidemic is therefore assumed to follow a left-truncated gamma distribution:

$$R_i - I_i \sim \mathcal{TG}(\alpha,\gamma,c), \tag{S5}$$

where I_i and R_i are respectively the infection and removal times for individual i. The density of the truncated gamma distribution is parameterised as:

$$f^{+}(R_i - I_i;\alpha,\gamma,c) = \frac{\gamma^{\alpha}}{\Gamma(\alpha,\gamma c)}(R_i - I_i)^{\alpha-1}\exp\left(-\gamma(R_i - I_i)\right)\mathbb{I}_{R_i - I_i > c}, \tag{S6}$$

where $\Gamma(\alpha,\gamma c) = \int_{\gamma c}^{\infty}\exp(-x)x^{\alpha-1}dx$ and $\mathbb{I}_{R_i-I_i>c}$ is the indicator function giving 1 if $R_i - I_i > c$ and 0 otherwise.

However we assume a slightly different version for simulated data with gamma distribution for the infectious period

$$R_i - I_i \sim \text{Ga}(\alpha,\gamma), \tag{S7}$$

where α and γ are respectively shape and rate parameters with probability density function defined as

$$f(R_i - I_i;\alpha,\gamma) = \frac{\gamma^{\alpha}}{\Gamma(\alpha)}(R_i - I_i)^{\alpha-1}\exp\left(-\gamma(R_i - I_i)\right)\mathbb{I}_{R_i - I_i > 0}; \tag{S8}$$

$\Gamma(\alpha) = \int_{0}^{\infty}\exp(-x)x^{\alpha-1}dx$ being the gamma function.

1.2 Likelihood

Before moving into the inferential framework for this process-based model, we need to write down the likelihood. The model as described above comprises two processes: infection and removal. Therefore the likelihood of the model can be written as

$$L(\boldsymbol{R}, \boldsymbol{I}; \boldsymbol{\theta}) \propto L_1 \times L_2 \tag{S9}$$

where L_1 and L_2 are the information coming from the infection and removal part of the likelihood respectively, and $\boldsymbol{\theta}$ is the vector of model parameters.

To explain the meaning of Equation (S9) in detail we introduce more notation. Let n_I be the total number of infections and v the first infected individual in the population. We denote by S the total person-to-person infectious pressure during the course of the epidemic. This is the case when we consider that an infection happen only when the total pressure exerted on a susceptible by the infectives is bigger than its threshold (Sellke, 1983). Therefore, we have

$$\begin{aligned} S &= \sum_{i=1}^{n_I}\sum_{j=1}^{N} \beta_{ij}((R_i \wedge I_j) - (I_i \wedge I_j)) \\ &= \beta_0 A \end{aligned}$$

where $A = \sum_{i=1}^{n_I}\sum_{j=1}^{N} h_{ij}((R_i \wedge I_j) - (I_i \wedge I_j))$. The infection process is actually a time-dependent Poisson process and S represents the fact there is no event happening between event times.

Hence the information coming from the infection part in the likelihood can be written as

$$L_1 = \prod_{i=1, i\neq v}^{n_I} \left(\sum_{j \in \mathcal{Y}_i} \beta_{ji} \right) \times \exp\{-S\} \tag{S10}$$

where $\mathcal{Y}_i = \{j : I_j < I_i < R_j\}$. The set $\mathcal{Y}_i$ considers all the infectious individuals exerting pressure on i at the time it became infected. It remains to consider the information coming from the removal part in the likelihood. Assuming that there are n_R removed individuals in the population, this can be written as

$$L_2 \propto \gamma^{\alpha n_R} \exp\left\{ -\gamma \sum_{i=1}^{n_R} (R_i - I_i) \right\} \prod_{i=1}^{n_R} \frac{(R_i - I_i)^{\alpha - 1}}{\Gamma(\alpha, \gamma c)}. \tag{S11}$$

using the probability density function of the truncated gamma distribution defined in (S6). In the case of simulated data, L_2 is obtained by the product of the probability density function of the gamma distribution as defined in (S8).

The likelihood of the model can then be obtained by multiplying Equations L_1 and L_2.

1.3 Bayesian inference

Data available from disease outbreaks are usually the times of detection or removal of the individuals. The infection times are regularly unknown unless some diagnostic tests are available leading to some knowledge of when the infections might have occurred. But in general, no information is available on the infection times. We assume that the infection times are not observed meaning that the likelihood in (S9) is obtained

using data augmentation techniques, hence includes missing data. The Bayesian framework is then adopted as it provides natural approach for handling missing data problems along with the computational tool Markov Chain Monte Carlo (MCMC) methods (O'Neill and Roberts, 1999; Jewell et al., 2009; Gamado et al., 2014).

The joint posterior distribution of the model parameters given the data is can be written as

$$\pi(\boldsymbol{\theta}|\boldsymbol{y}) \propto L(\boldsymbol{y};\boldsymbol{\theta})\pi(\boldsymbol{\theta}), \tag{S12}$$

where $\pi(\boldsymbol{\theta})$ is the joint prior distribution on the model parameters and $L(\boldsymbol{y};\boldsymbol{\theta})$ is the likelihood function with $\boldsymbol{y}$ representing the observed and unobserved data.

By defining gamma prior on β_0 ($\beta_0 \sim \text{Ga}(\lambda_{\beta_0}, \nu_{\beta_0})$), we obtain the full posterior conditional distribution:

$$\beta_0|\gamma, \tau, \boldsymbol{R}, \boldsymbol{I} \sim \text{Ga}(n_I + \lambda_{\beta_0} - 1, \nu_{\beta_0} + A) \tag{S13}$$

meaning that the gamma distribution is a conjugate prior β_0. The property of conjugacy is also obtained for γ if we assume a gamma distribution for the infectious period as in Equation (S7). The full posterior conditional distribution of γ is given by

$$\gamma|\beta_0, \tau, \boldsymbol{R}, \boldsymbol{I} \sim \text{Ga}\left(\alpha n_R + \lambda_\gamma, \nu_\gamma + \sum_{i=1}^{n_R}(R_i - I_i)\right), \tag{S14}$$

when assuming $\gamma \sim \text{Ga}(\lambda_\gamma, \nu_\gamma)$ prior. However, when assuming the left-truncated gamma distribution in (S5) for the infectious period, the conjugacy property does not apply and we update γ using Metropolis-Hastings algorithms (Metropolis et al., 1953). The infection times, τ, d and α are also updated through Metropolis-Hastings scheme since we are not aware of existing closed forms for their posterior distributions. The model parameters τ, d and α (also γ if necessary) are updated using a random walk scheme: For the parameter τ for instance, we propose a new value $\tau' = \tau + U(-a, a)$, where $U(-a, a)$ is a random variate drawn from the uniform distribution. Note that the choice of a is important for facilitating convergence. The proposed value τ' is accepted with probability

$$A = \min\left(\frac{L(\boldsymbol{y};\boldsymbol{\theta}')}{L(\boldsymbol{y};\boldsymbol{\theta})}, 1\right),$$

where $\boldsymbol{\theta}'$ is the vector of model parameters with τ replaced by τ'. If τ' is not accepted, we retain the current value of τ.

The infection times are updated using a simple non-centering scheme (Neal and Roberts, 2005; Papaspiliopoulos et al., 2007). For an individual i we propose a new infection time I_i' bases on the assumption of the removal process $R_i - I_i' \sim \mathcal{TG}(\alpha, \gamma, c)$. We accept I_i' with probability

$$\frac{L(\boldsymbol{R}, \boldsymbol{I'};\boldsymbol{\theta})}{L(\boldsymbol{R}, \boldsymbol{I};\boldsymbol{\theta})} \times \frac{f^+(R_i - I_i; \alpha, \gamma, c)}{f^+(R_i - I_i'; \alpha, \gamma, c)},$$

where $\boldsymbol{I'}$ is the vector of infection times with I_i replaced by I_i'. The same steps are applied for the case of gamma distribution considered for the infectious period with f^+ replaced by f.

1.4 Model choice

1.4.1 Latent residuals method

"To innovate a statistically sound framework for assessing stochastic spatio-temporal models, which can be readily implemented as an addendum to a Bayesian analysis and which avoids the sensitivity and complexity of Bayesian model assessment" (Lau et al., 2014) introduce the use of latent residuals. Bayesian latent residuals are the unobserved, independent, uniform random variables that conform with the data generation process. The root of the concept comes from the posterior predictive p-values proposed by Meng (1994). It has been since extended by Gibson et al. (2006) and Streftaris and Gibson (2012), the latter who use it to assess the threshold of individuals in the Sellke construction (Sellke, 1983). It is an illustration of the concept of generalised residuals proposed in Cox and Snell (1968) and the scheme is equivalent to non-centered parameterisation (Papaspiliopoulos et al., 2007).

In this particular case where the tests are designed for detecting mis-specification of spatial transmission kernel, there is a need to re-construct the infection links ("who infects who") from which the latent residuals are obtained. These are the infection-link residuals and more details can be read in Lau et al. (2014). Anderson-Darling hypothesis test (Lewis, 1961) or Kolmogorov-Smirnov hypothesis test (Marsaglia et al., 2003) can then be used to discern evidence against modelling assumptions. We emphasise that the latent residuals method used frequentist framework to analyse outputs (residuals) from Bayesian model fitting and the statistical evidences are based on p-values.

We then move on to simulate bigger epidemics i.e. the number of removals are increased and re-start the process described above until the p-values clearly give us evidence of selecting one model over others i.e the preferred kernel transmission function. The corresponding epidemic size from which a model is selected gives us the size needed to select and assess the correct transmission function.

1.4.2 Computation of the Deviance Information Criteria (DIC)

We also use a purely Bayesian model selection tool (DIC) to compare with the latent residuals in terms of results and practicality in implementation. The main difference between the DIC computed here and the DIC provided by software such as BUGS (Lunn et al., 2000; Thomas et al., 2006) is that we are in the presence of data augmented likelihood as the observation process is incomplete. We adopt two of the DICs described in Celeux et al. (2006) and compute them to select models, namely

$$DIC_1 = -4\mathbb{E}_{\boldsymbol{\theta},\boldsymbol{X}}\left[\log\left(f(\boldsymbol{y},\boldsymbol{X}|\boldsymbol{\theta})\right)|\boldsymbol{y}\right] + 2\mathbb{E}_{\boldsymbol{X}}\left[\log\left(f\left(\boldsymbol{y},\boldsymbol{X}|\mathbb{E}_{\boldsymbol{\theta}}\left[\boldsymbol{\theta}|\boldsymbol{y},\boldsymbol{X}\right]\right)\right)|\boldsymbol{y}\right] \tag{S15}$$

and

$$DIC_2 = -4\mathbb{E}_{\boldsymbol{\theta},\boldsymbol{X}}\left[\log\left(f(\boldsymbol{y}|\boldsymbol{X},\boldsymbol{\theta})\right)|\boldsymbol{y}\right] + 2\mathbb{E}_{\boldsymbol{X}}\left[\log\left(f\left(\boldsymbol{y}|\boldsymbol{X},\hat{\boldsymbol{\theta}}(\boldsymbol{y},\boldsymbol{X})\right)\right)|\boldsymbol{y}\right], \tag{S16}$$

where $\boldsymbol{X}$ and $\boldsymbol{y}$ represent the unobserved and observed data respectively and $\hat{\boldsymbol{\theta}}(\boldsymbol{y},\boldsymbol{X})$ is a posterior point estimate (posterior median here). The subtle difference between the two quantities is that $f(\boldsymbol{y},\boldsymbol{X}|\boldsymbol{\theta})$ denote the full likelihood i.e the contribution to the likelihood from both the observation and process models and $f(\boldsymbol{y}|\boldsymbol{X},\boldsymbol{\theta})$ is the partial likelihood i.e the contribution to the likelihood from the observation only.

The two DICs present two terms in their respective formula that need to be computed separately. While inferring the model parameters through MCMC, we make sure to store at each iteration the full and partial log-likelihoods that correspond to the parameter values and latent variables. Taking for each the expected values of the full and partial log-likelihoods times -4 give the left hand terms in each of the DICs. Considering now the posterior density of our parameter vector, we take a point estimate (mean for DIC_1,

any for DIC_2) and re-run the full log-likelihood or partial log-likelihood computation for each MCMC iteration using the stored latent variables and the posterior point estimate. We again take the expected values of the obtained full and partial log-likelihoods separately and multiply by 2. The sum of the two expected full log-likelihood and partial log-likelihood give respectively DIC_1 and DIC_2.

2 STATISTICAL INFERENCE

2.1 Single simulated data

2.1.1 Inference

Single data was simulated using either K_1 (simulation study 1a) with $\beta_0 = 0.35$, $h_{ij} = \exp\{-0.008\rho(i,j)\}$ or K_2 (simulation study 1b) with $\beta_0 = 400$, $h_{ij} = \frac{1}{1+\left(\frac{\rho(i,j)}{1.5}\right)^2}$. The infectious period follows a $\mathrm{Ga}(5,5)$ distribution, departing from the non-realistic assumption of exponentially distributed infectious period. Summary statistics of the posterior estimates obtained for each simulated data are provided in Tables S1 and S2 respectively when fitting the kernels K_1 and K_2.

	mean	sd	2.5%	50%	97.5%
β_0	0.396	0.153	0.169	0.372	0.761
γ	6.251	2.323	2.638	5.881	11.631
τ	0.00771	0.00125	0.00540	0.00767	0.01031
α	4.907	1.848	1.986	4.673	9.190

Table S1 Posterior estimates for β_0, γ, τ and α when fitting K_1 to simulation study 1a; non-informative priors and $\mathrm{Gamma}(5,1)$ prior on α

	mean	sd	2.5%	50%	97.5%
β_0	714.576	724.469	59.319	470.882	2809.770
γ	7.931	2.745	3.298	7.725	13.429
τ	2.016	0.188	1.639	2.011	2.411
α	5.567	2.091	2.228	5.367	9.782
d	2.237	1.001	0.699	2.076	4.717

Table S2 Posterior estimates for β_0, γ, τ, α and d when fitting K_2 to simulation study 1b; non-informative priors and $\mathrm{Gamma}(5,1)$ prior on α

The posterior densities are plotted on Figures S1 and S3 for the K_1 and K_2 parameters respectively. The prior distributions are superimposed in red-dotted lines with the posterior distributions and the true parameter values are indicated in green vertical solid lines. All the true parameter values fall within the 95% credible intervals. The shape of the gamma distribution α requires some prior knowledge as many previous studies show (Streftaris and Gibson, 2004; Kypraios, 2007). The posterior samples were assessed and no evidence of lack of convergence was detected as shown by the auto-correlation functions plotted in Figures S2 and S4.

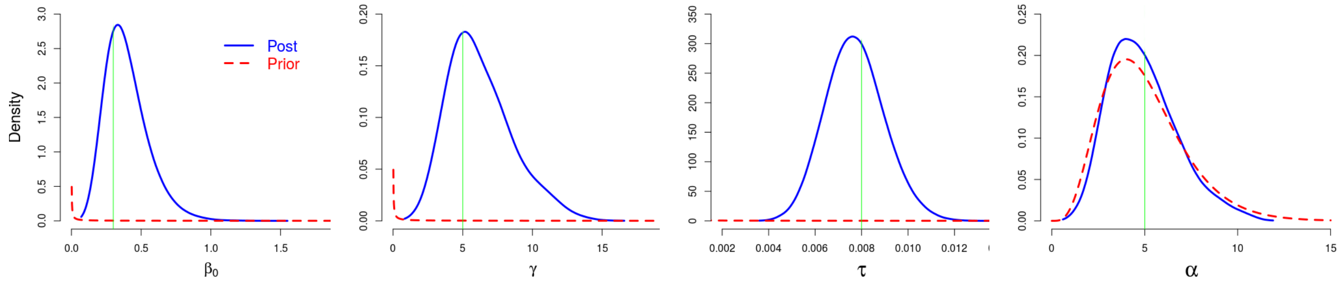

(a) β_0 **(b)** γ **(c)** τ **(d)** α

Figure S1. Posterior densities of the model parameters β_0 (a), γ (b), τ (c), and α (d), when fitting K_1 to simulation study 1a. The true parameter values are represented in green vertical lines

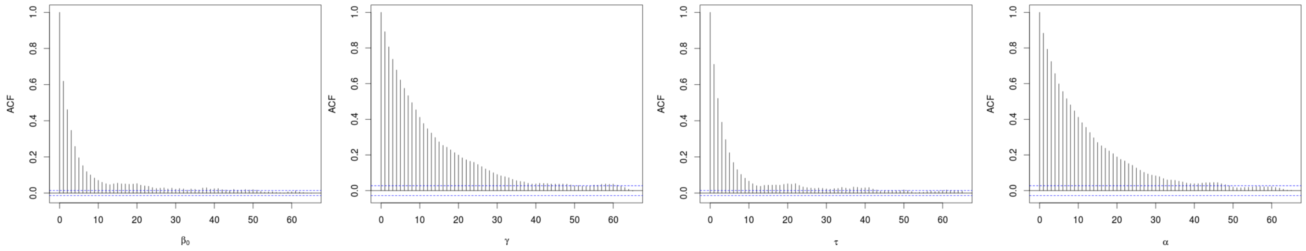

(a) β_0 (b) γ (c) τ (d) α

Figure S2. Auto-correlation functions (ACF) of the model parameters β_0 (a), γ (b), τ (c), and α (d), when fitting K_1 to simulation study 1a.

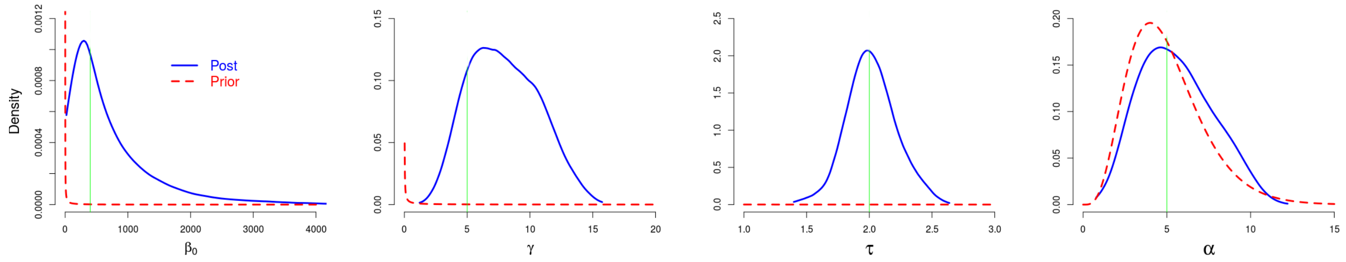

(a) β_0 (b) γ (c) τ (d) α

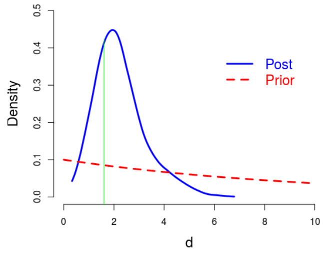

(e) d

Figure S3. Posterior densities of the model parameters β_0 (a), γ (b), τ (c), α (d) and d (e), when fitting K_2 to simulation study 1b. The true parameter values are represented in green vertical lines

2.1.2 Model choice

The latent residuals provide p-values at each MCMC simulations and their distributions provide evidence of model assessment and selection.

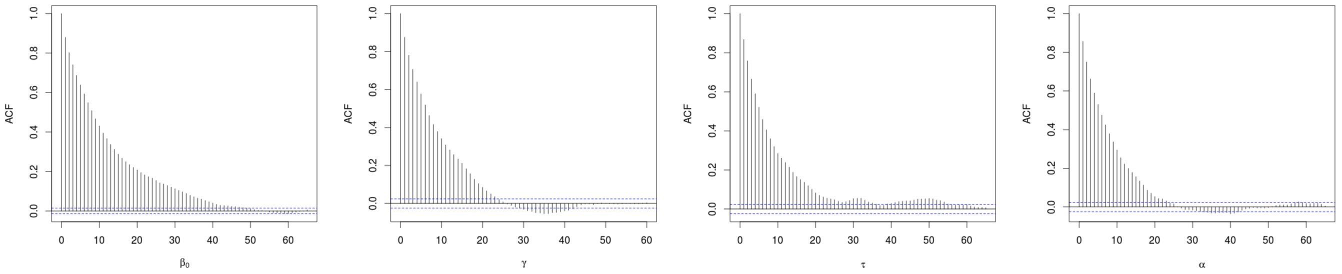

(a) β_0 (b) γ (c) τ (d) α

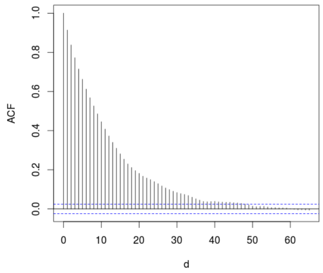

(e) d

Figure S4. Auto-correlation functions (ACF) of the model parameters β_0 (a), γ (b), τ (c), α (d), and d (e), when fitting K_2 to simulation study 1b

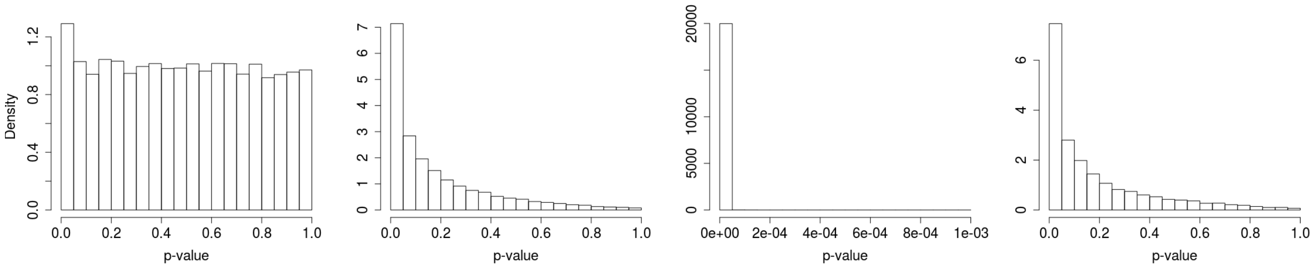

(a) K_1 (b) K_2 (c) K_3 (d) K_4

Figure S5. Posterior distributions of p-values testing the sets of posterior samples of infection-link residuals (ILR) for simulation study 1a. The kernels fitted are K_1 (a), K_2 (b), K_3 (c) and K_4 (d).

Models with distributions of p-values clustered around small values (here less than 5%) are evidence of non-agreement with the data as we can see with Figures S5 (b), (c) and (d) when the data was simulated using K_1 and Figure S6 (a) and (c) when the true kernel was K_2. When data came from K_2, K_4 also seems to conform with it and it is not obvious to distinguish between the two kernels. However, we found that it is related to the population density as shown in Section 3.

2.1.3 Final size distributions

Final size distributions of the simulated epidemics based on the inferred model parameters are presented in Figures S7 and S8 for true models K_1 and K_2 respectively. As expected, these distributions are bimodal (Andersson and Britton, 2000).

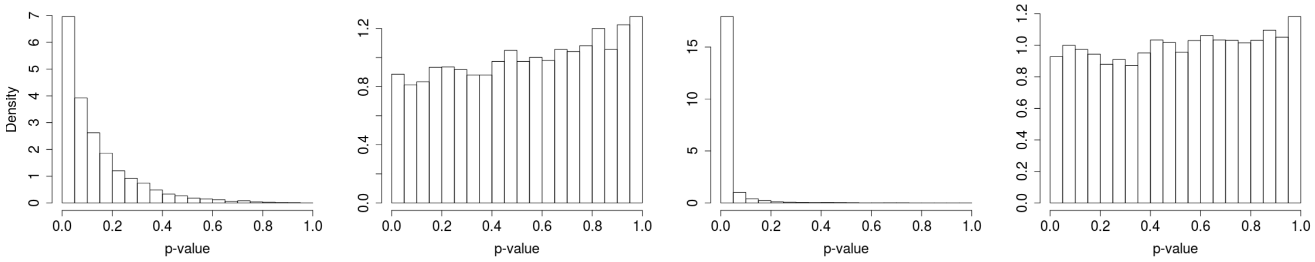

(a) K_1 (b) K_2 (c) K_3 (d) K_4

Figure S6. Posterior distributions of p-values testing the sets of posterior samples of infection-link residuals (ILR) for simulation study 1b. The kernels fitted are K_1 (a), K_2 (b), K_3 (c) and K_4 (d).

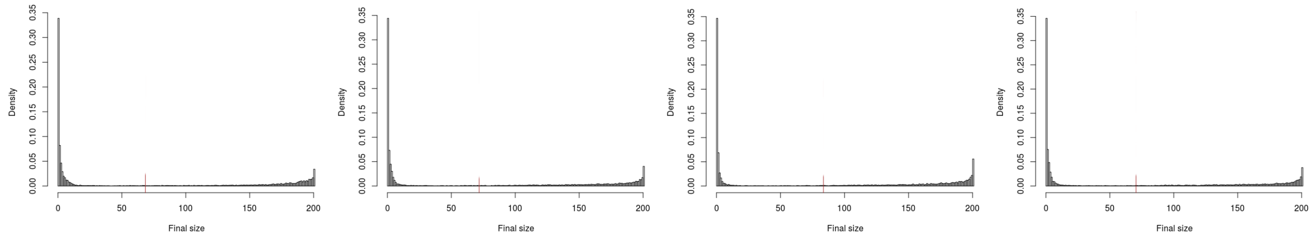

(a) Final size under K_1 (b) Final size under K_2 (c) Final size under K_3 (d) Final size under K_4

Figure S7. Epidemic final size distributions under K_1, K_2, K_3 and K_4, when using inferred parameters from simulation study 1a. The vertical line is the mean final size.

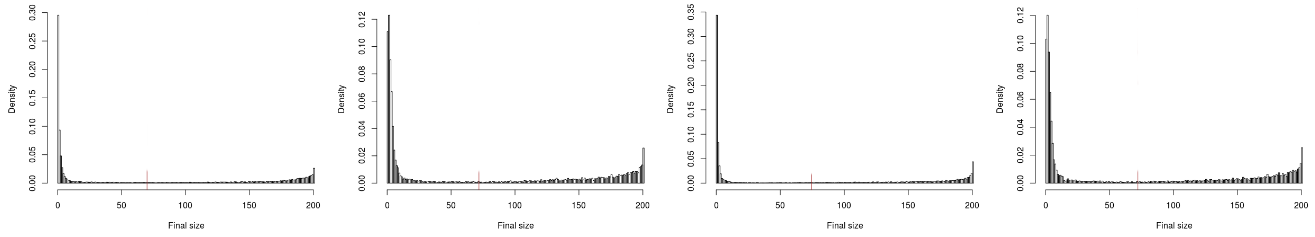

(a) Final size under K_1 (b) Final size under K_2 (c) Final size under K_3 (d) Final size under K_4

Figure S8. Epidemic final size distributions under K_1, K_2, K_3 and K_4, when using inferred parameters from simulation study 1b. The vertical line is the mean final size.

2.1.4 Risk maps

The inferred parameters appear to be different but the most appealing evidences in the choice of kernels for policy purposes are risk maps since they provide probabilities of infection for farms. Such maps can then be used to target high risk areas for disease control. Posterior risk maps based on simulation studies 1a and 1b data are plotted on Figures S9 and S10 respectively.

The posterior predictive risks obtained based on the simulation study 1b also show that K_3 provides the highest risk profile for the farms and similar risks for all the farms under K_2 and K_4. The risk profile in this case is smaller under K_2 and K_4 compared to K_1.

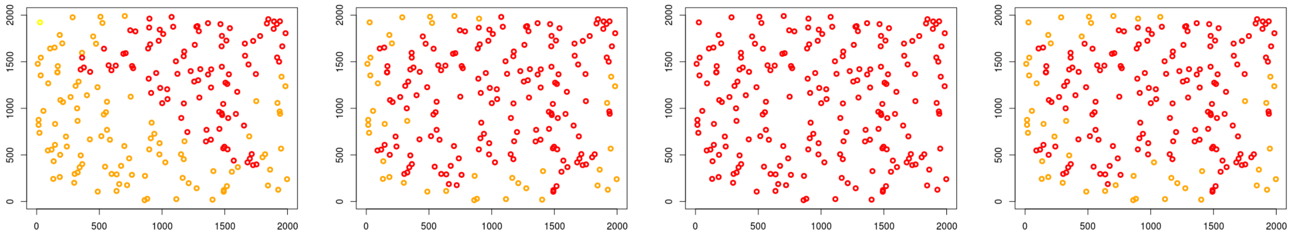

(a) K_1 (b) K_2 (c) K_3 (d) K_4

Figure S9. Comparison of the risk maps using K_1 (a), K_2 (b), K_3 (c) and K_4 (d) at time $t = 5$ days, corresponding to a length of time sufficient to capture the early phase and small scale epidemics' behaviour, based on the population in a square of sides $[0, 2000]$ km on the x and y axis of simulation study 1a.

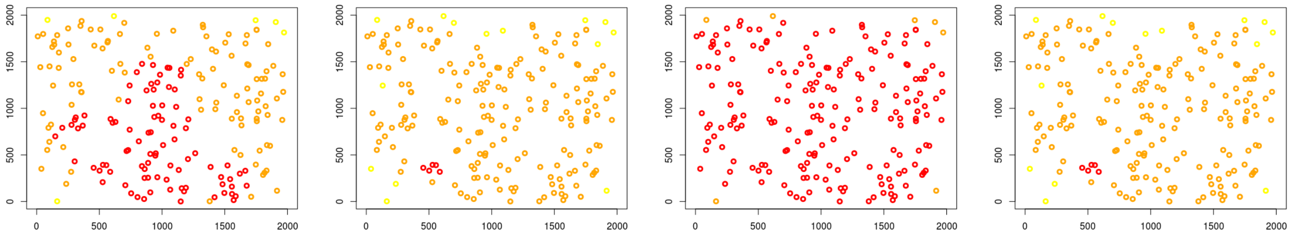

(a) K_1 (b) K_2 (c) K_3 (d) K_4

Figure S10. Comparison of the risk maps using K_1 (a), K_2 (b), K_3 (c) and K_4 (d) at time $t = 5$ days, corresponding to a length of time sufficient to capture the early phase and small scale epidemics' behaviour, based on the population in a square of sides $[0, 2000]$ km on the x and y axis of simulation study 1b.

2.2 Simulation studies

The coverage rate i.e. the proportion of the time the true parameter values fall within the 95% credible intervals are recorded in Tables S3 and S4 when data are simulated using K_1 and K_2 respectively.

	$[6,10]$	$[11,15]$	$[16,20]$	$[21,25]$	$[26,30]$	$[31,35]$	$[36,40]$	$[41,45]$
β_0	96.67	96.67	96.67	93.33	93.33	93.33	93.33	96.67
γ	100	96.67	100	100	100	100	100	100
τ	93.33	93.33	100	93.33	93.33	86.67	90.00	96.67
α	100	96.67	100	100	100	100	100	100

Table S3 Coverage rates in $\%$ from simulation studies of the parameters as a function of epidemic sizes when the data simulations are carried using K_1.

	$[6,10]$	$[11,15]$	$[16,20]$	$[21,25]$	$[26,30]$	$[31,35]$	$[36,40]$	$[41,45]$
β_0	96.67	96.67	100	96.67	96.67	96.67	93.33	96.67
γ	100	100	100	100	96.67	100	100	100
τ	96.67	100	96.67	96.67	93.33	96.67	93.33	96.67
d	100	96.67	96.67	96.67	93.33	96.67	96.67	96.67
α	100	100	100	100	96.67	100	100	100

Table S4 Coverage rates in $\%$ from simulation studies of the parameters as a function of epidemic sizes when the data simulations are carried using K_2.

The coverage rates are expected to be 95% which is approximately the case generally for most parameters. However, the parameters of the infectious period distributions are often higher. One explanation would be the informative prior on one of the parameters as other authors have assumed in the past (Streftaris and Gibson, 2004; Kypraios, 2007).

2.3 All methods selecting the same kernel

Given that not all the selection tools agree with each other, we look specifically at the cases where all methods select the same model. When all the methods select the same model, we evaluate the proportions that such model is the correct as a function of the epidemic sizes. As visible in Figures S11 (a) and (c), for epidemic sizes greater than 10, there is a proportion of 100% of selecting the correct model when all model choice tools select that model. Figure S11 (b) does not show a clear pattern and this is due to the fact that the selection methods find it difficult to separate K_2 and K_4. A clear indication is that when ignoring K_4 in Figure S11 (c), we obtain a 100% of choosing the correct model when all the methods agree.

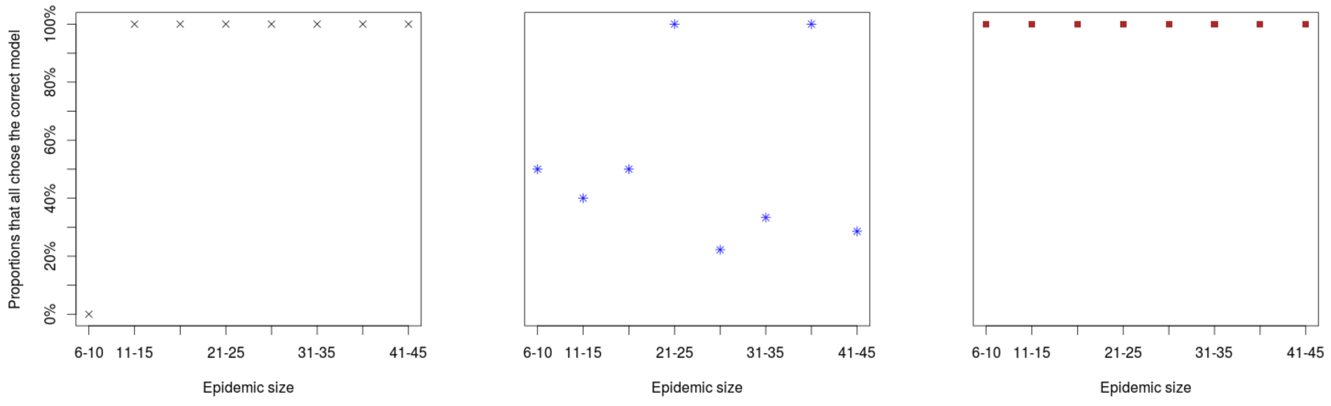

(a) (b) (c)

Figure S11. Proportions that all methods select the correct model as a function of the epidemic size when all data is simulated using K_1 (a) and when the data is simulated using K_2 (b) but with K_4 ignored (c)

2.4 Posterior estimates for the CSF data

In Figure S12, we superimpose the posterior distributions of the common parameters from the 4 kernels K_1-K_4. The infection rate of K_1 is the smallest among all the kernels with a small overlap with the other densities plots. The rate of K_1 is confined to an area while the infection rates of K_2, K_3 and K_4 are more spread out with a very long right-tail as visible in Figure S12 (a). The parameter d behaves in opposite direction to β_0 by being more spread out with very long right-tailed for K_1. Note that d for the K_1 is equivalent to $1/\tau$ following the notation in Equations (S1)-(S4) and is plotted in Figure S12 (b).

Not surprisingly the parameters of the distributions of the infectious period look very similar since that part is identical for all 4 models. This is also confirmed by the mean posterior distribution of the left-truncated gamma distribution of the infectious period in Figure S13.

The posterior medians and their corresponding 95% credible intervals of the kernel transmission function are plotted on a log-scale under the four different kernels in Figure S14.

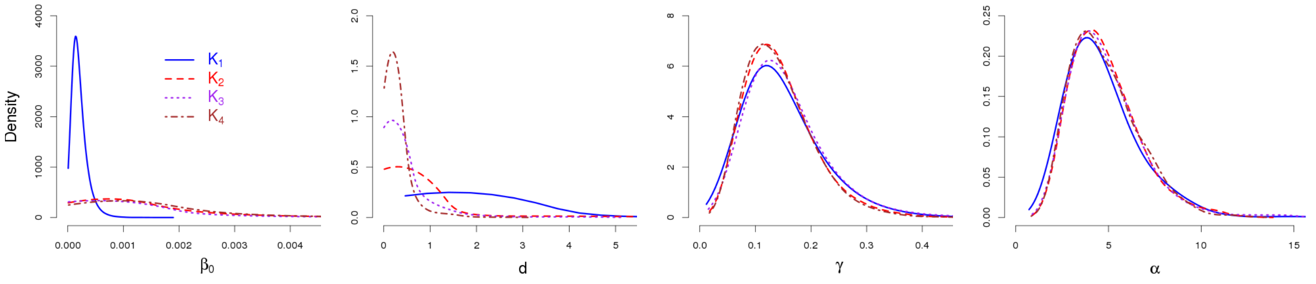

(a) β_0 (b) d (c) γ (d) α

Figure S12. Superimposition of the posterior distributions of the common parameters β (a), d (b), γ (c) and α (d) from K_1 (blue solid line), K_2 (red dashed line), K_3 (purple dotted line) and K_4 (brown dot-dashed line) using the CSF data

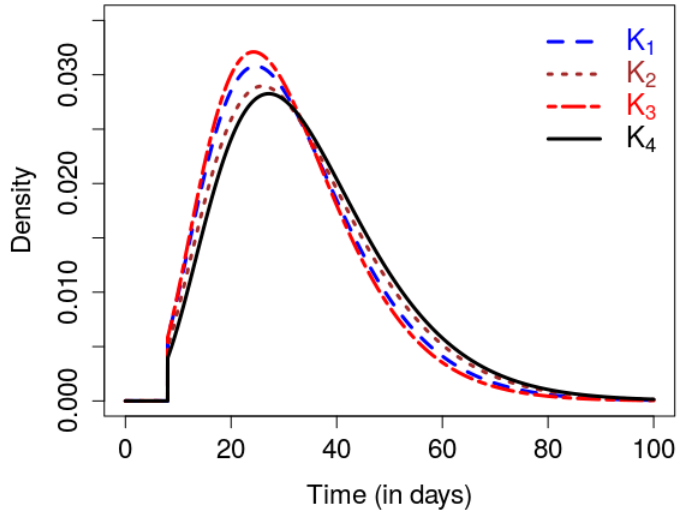

Figure S13. Densities of the left-truncated gamma distribution at the mean posterior estimates of the parameters under K_1-K_4

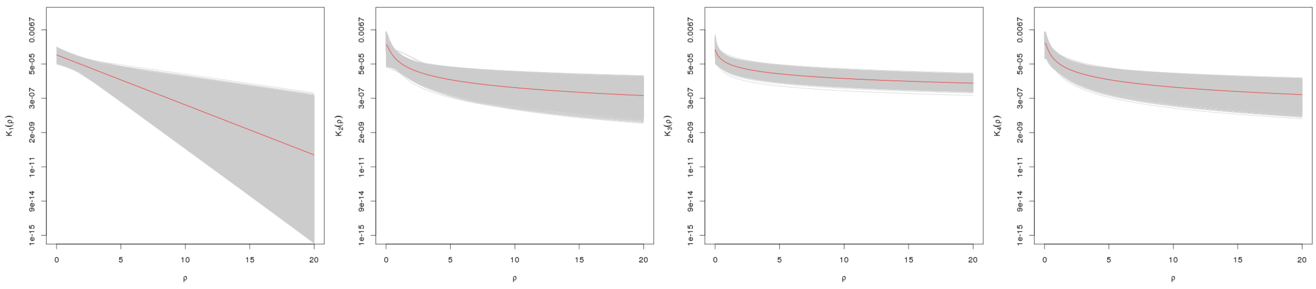

(a) K_1 (b) K_2 (c) K_3 (d) K_4

Figure S14. Posterior estimates of the transmission kernel functions β_{ij} under K_1 (a), K_2 (b), K_3 (c), and K_4 (d) on a log scale and assuming that individuals are infected at least 8 days before getting detected. The solid line represents the posterior median and the ribbon is the 95% credible interval for each of the four kernels.

Summary statistics of the posterior estimates under the four different kernels are shown in Table S5. The inferred common parameters of the various kernels appear to be different while the posterior distributions of parameters of the left-truncated gamma distributions were very similar. This is not surprising since the various models assume the same distribution for the infectious period and differ only from the kernel transmission functions.

			K_1		
	mean	sd	2.5%	50%	97.5%
β_0	2.06×10^{-4}	1.35×10^{-4}	4.62×10^{-5}	1.74×10^{-4}	5.54×10^{-4}
γ	1.50×10^{-1}	7.63×10^{-2}	5.06×10^{-2}	1.36×10^{-1}	3.28×10^{-1}
τ	7.59×10^{-1}	2.92×10^{-1}	2.66×10^{-1}	7.30×10^{-1}	1.40×10^{0}
α	4.70×10^{0}	2.03×10^{0}	1.92×10^{0}	4.33×10^{0}	9.41×10^{0}
			K_2		
	mean	sd	2.5%	50%	97.5%
β_0	1.18×10^{-3}	1.60×10^{-3}	3.81×10^{-5}	7.25×10^{-4}	5.49×10^{-3}
γ	1.39×10^{-1}	6.11×10^{-2}	4.99×10^{-2}	1.29×10^{-1}	2.89×10^{-1}
d	8.30×10^{-1}	1.38×10^{0}	4.08×10^{-2}	2.79×10^{-1}	5.53×10^{0}
τ	1.84×10^{0}	7.61×10^{-1}	9.41×10^{-1}	1.71×10^{0}	3.80×10^{0}
α	4.63×10^{0}	1.73×10^{0}	2.00×10^{0}	4.38×10^{0}	8.70×10^{0}
			K_3		
	mean	sd	2.5%	50%	97.5%
β_0	9.07×10^{-4}	1.59×10^{-3}	4.85×10^{-5}	4.07×10^{-4}	4.86×10^{-3}
γ	1.57×10^{-1}	7.62×10^{-2}	5.39×10^{-2}	1.43×10^{-1}	3.43×10^{-1}
d	3.54×10^{-1}	5.85×10^{-1}	1.05×10^{-2}	1.38×10^{-1}	1.98×10^{0}
α	4.88×10^{0}	2.03×10^{0}	2.05×10^{0}	4.52×10^{0}	9.80×10^{0}
			K_4		
	mean	sd	2.5%	50%	97.5%
β_0	1.34×10^{-3}	1.41×10^{-3}	1.01×10^{-4}	9.25×10^{-4}	5.05×10^{-3}
γ	1.42×10^{-1}	6.19×10^{-2}	5.09×10^{-2}	1.33×10^{-1}	2.89×10^{-1}
d	2.59×10^{-1}	2.83×10^{-1}	3.50×10^{-2}	1.78×10^{-1}	1.14×10^{0}
τ	1.61×10^{0}	3.49×10^{-1}	9.96×10^{-1}	1.58×10^{0}	2.38×10^{0}
α	4.79×10^{0}	1.84×10^{0}	2.04×10^{0}	4.49×10^{0}	8.99×10^{0}

Table S5 Posterior estimates for model parameters using kernels $K_1 - K_4$; non-informative priors and Gamma$(4, 1)$ prior on α, assuming that individuals are infected at least 8 days before getting detected.

The posterior distributions of p-values when fitting the CSF data and using the latent residuals method for selecting models are plotted in Figure S15.

The p-values show greater evidence against K_3 and K_1 by producing a higher frequency of small values, followed by K_4. K_2 is selected since it produces less evidence of disagreement with the data despite not being ideal.

One of the advantages of using Bayesian inference on this type of datasets is the possibility of inferring the latent variables, particularly the infection times in this context. The posterior estimates of the infection times are summarised in the form of box plots in Figure S16 for all the 16 cases. Independent estimates through contact tracing procedures of the infection times were carried out during control activities and we mark those estimates in red on the box plots. Despite some of the contact tracing inferred times fall in the

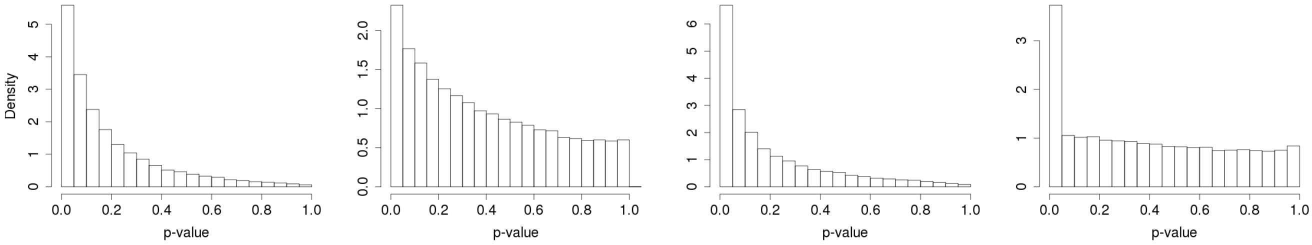

(a) K_1 (b) K_2 (c) K_3 (d) K_4

Figure S15. Posterior distributions of p-values testing the sets of posterior samples of infection-link residuals (ILR) for the CSF data of 16 infected cases in Norfolk (East Anglia). The kernels fitted are K_1 (a), K_2 (b), K_3 (c) and K_4 (d).

tail of the distributions, all of them fall well within the range of our posterior estimates except for the last farm. Our estimates show that it is the most probable last infected farm but the contact tracing attributes a very late infection to it. It is worth noting that we considered the first detected time as being 0, reason why the y-axis for infection times show negative times.

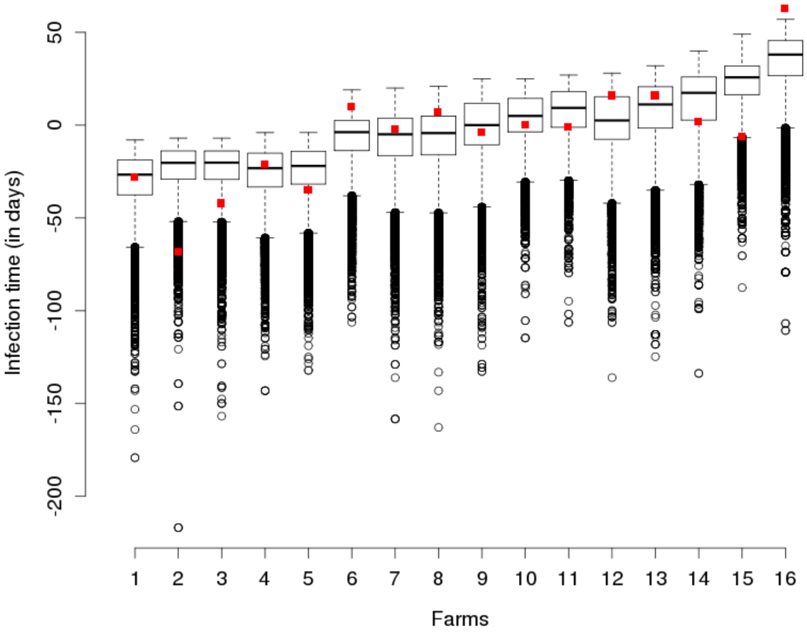

Figure S16. Distribution of the infection times as a box plot for each infected farm with the believed infection times through contact tracing in red. Note the re-scaling of time here where we assume that the first detection happens at time 0.

2.5 Final size distributions based on the CSF data

Final size distributions of the simulated epidemics based on the posterior distributions obtained under each model are plotted in Figure S17 on a log-scale. The final size distribution is bimodal with some epidemic ending very quickly and others affecting a good proportion of the population with rare medium epidemic size.

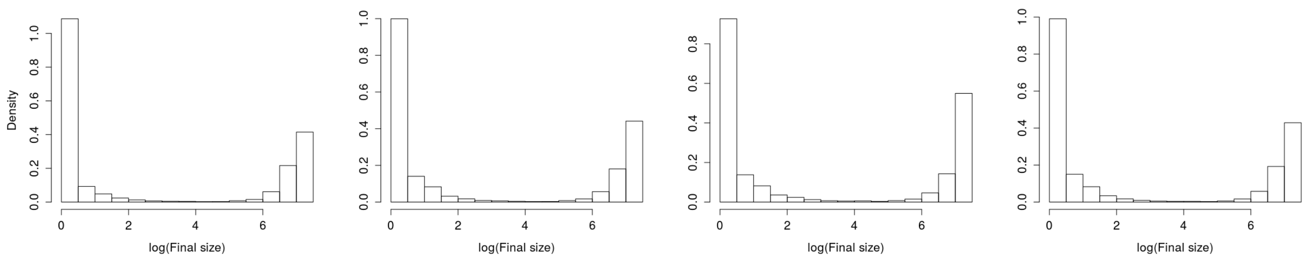

(a) K_1 (b) K_2 (c) K_3 (d) K_4

Figure S17. Epidemic final size distributions on a log-scale under K_1, K_2, K_3 and K_4, when using inferred parameters from the CSF epidemic.

3 EFFECT OF THE POPULATION DENSITY ON THE KERNELS AND MODEL SELECTION

3.1 Simulation study on low population density

Simulation studies performed as a function of the epidemic size shows that when K_2 is used as baseline,

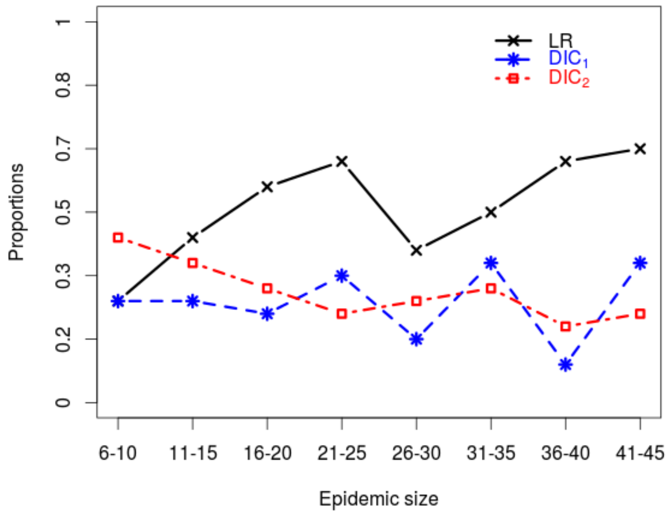

Figure S18. Proportions of correctly selecting the right model using latent residuals (LR), DIC$_1$ and DIC$_2$ in a simulation study using K_2 as baseline and considering all four kernels

K_2 and K_4 are difficult to dissociate. Although the latent residuals seem to select better K_2 than the two DICs (Figure S18), the effect of the epidemic size is not clear and the rate of selecting the right kernel, although not negligible still needs to be higher for a good level of confidence. This is due to a very low average population density of 5×10^{-5} farms per unit area for the epidemic simulations as we illustrate in the following section.

3.2 Infection-link residuals applied to varying population densities

The shapes of K_2 and K_4 suggest that the two kernels would be more appropriate in the cases of diseases' spread that are particularly local. In the simulation studies carried in this manuscript, it appears that K_2

and K_4 are indissociable i.e. it is not clear if one of the two kernels is preferred over the other while it seems to be the case in the real data application. The effect of the two kernel transmission functions as a function of the population density is then studied. The number of individuals per unit area is first adopted as a measure of population density. Three scenarios are considered: Firstly, the number of individuals per unit area from the classical swine fever data is matched; followed by a ten times less dense population and finally a ten times more dense initial population at risk. Two simulations of disease spread are carried over each scenario with in fact not the population sizes increased but rather the areas are either reduced or increased to match the desired number of individuals per unit area. The epidemics are simulated assuming K_2 and inference is performed on the data which consist of the removal times of the infected sites together with the whole population locations. Model selection are performed using the latent residuals and the two DICs defined in Section 1.4. The posterior distributions of the p-values in Figure S19 show the effect of the population density on the dissociation of K_2 and K_4. More dense populations to the CSF data demonstrate a clear difference in the kernels while less dense populations were not able to select one kernel over the other. The single measurement of the proportions of p-values less than 5% in Table S6 simply confirms the overall distributions conclusions that p-values are well different when the population density increases while failing to select between the kernels in the case of small density area.

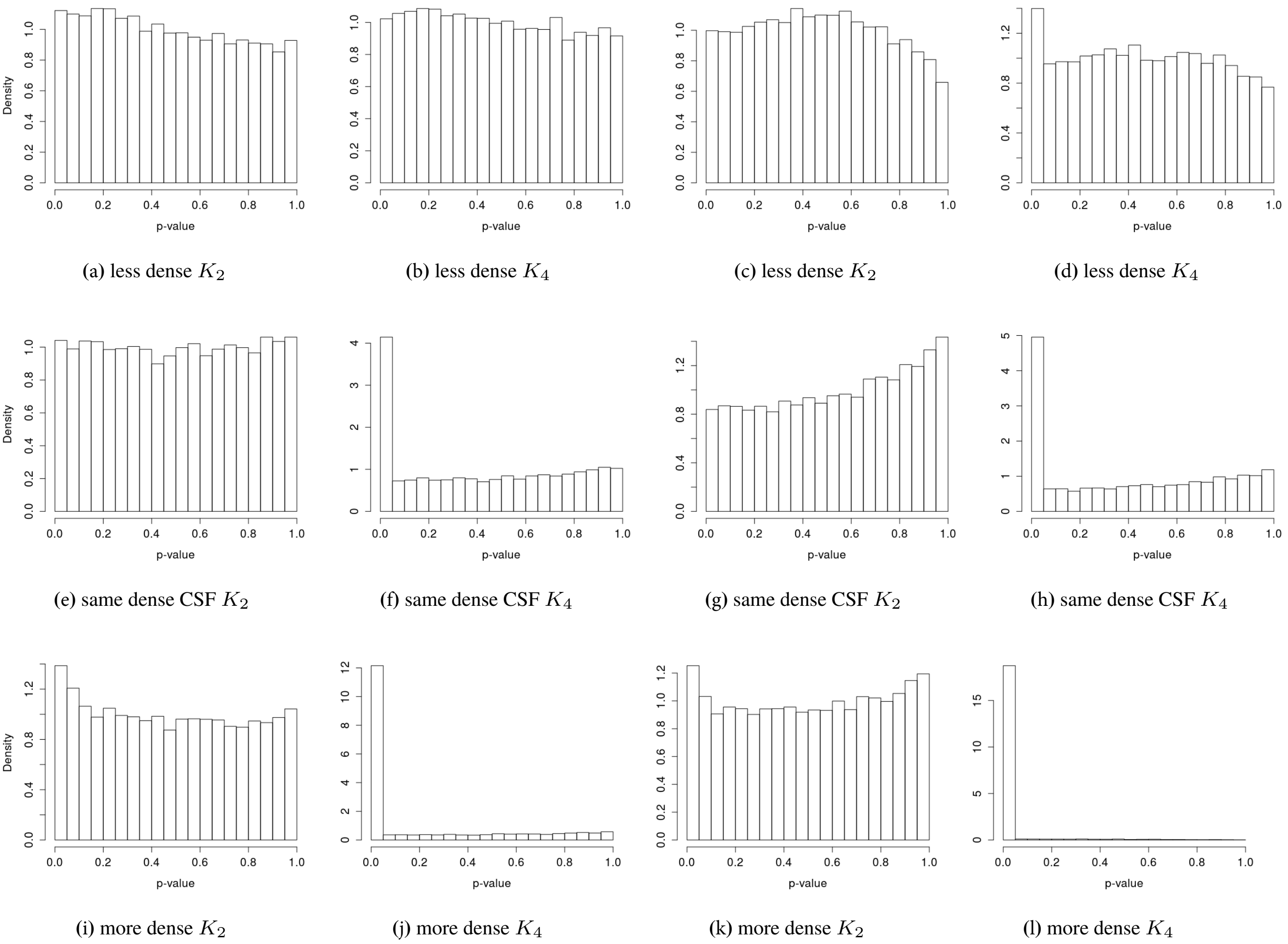

(a) less dense K_2 (b) less dense K_4 (c) less dense K_2 (d) less dense K_4

(e) same dense CSF K_2 (f) same dense CSF K_4 (g) same dense CSF K_2 (h) same dense CSF K_4

(i) more dense K_2 (j) more dense K_4 (k) more dense K_2 (l) more dense K_4

Figure S19. Distribution of p-values for selecting between K_2 and K_4 comparing cases of same average number of individuals per unit area with the CSF data, 10 times less and 10 times more individuals in the unit area on average compared to the CSF data

Kernels	$10\times$ less dense		dense as CSF		$10\times$ more dense	
$K_2(\%)$	5.6	4.9	5.2	4.2	6.9	6.2
$K_4(\%)$	5.1	6.9	20.7	24.7	60.8	92.4

Table S6 Proportions of p-values less than 5% (Pr$(p < 5\%)$ in $\%$) for selecting between K_2 and K_4 when comparing cases of same average number of individuals per unit area with the CSF data (middle); 10 times less (left) and 10 times more (right) individuals in the unit area on average compared to the CSF data.

REFERENCES

Andersson, H. and Britton, T. (2000). *Stochastic Epidemic Models and Their Statistical Analysis* (New York: Springer)

Celeux, G., Forbes, F., Robert, C. P., and Titterington, D. M. (2006). Deviance information criteria for missing data models. *Bayesian Analysis* 1, 651–674. doi:10.1214/06-BA122

Cox, D. R. and Snell, E. (1968). A general definition of residuals. *J. R. Stat. Soc. B* 30, 248–275

Dawson, P. M., Werkman, M., Brooks-Pollock, E., and Tildesley, M. J. (2015). Epidemic predictions in an imperfect world: modelling disease spread with partial data. *Proc. R. Soc. B* 282, 20150205. doi:10.1098/rspb.2015.0205

Gamado, K. M., Streftaris, G., and Zachary, S. (2014). Modelling under-reporting in epidemics. *Journal of Mathematical Biology* 69, 737–765. doi:10.1007/s00285-013-0717-z

Gibson, G., Otten, W., Filipe, J., Cook, A., Marion, G., and Gilligan, C. (2006). Bayesian estimation for percolation models of disease spread in plant populations. *Stat. Comput.* 16, 391–402. doi:10.1007/s11222-006-0019-z

Jewell, C. P., Keeling, M. J., and Roberts, G. O. (2009). Predicting undetected infections during the 2007 foot-and-mouth disease outbreak. *J. R. Soc. Interface* 6, 1145–1151. doi:10.1098/rsif.2008.0433

Keeling, M. J. and Rohani, P. (2007). *Modeling Infectious Diseases in Humans and Animals* (Princeton, NJ: Princeton University Press)

Kypraios, T. (2007). *Efficient Bayesian Inference for Partially Observed Stochastic Epidemics and A New Class of SemiParametric Time Series Models.* PhD thesis, Department of Mathematics and Statistics, Lancaster University, Lancaster

Lau, M. S. Y., Marion, G., Streftaris, G., and Gibson, G. (2014). New model diagnostics for spatio-temporal systems in epidemiology and ecology. *J. R. Soc. Interface* 11, 20131093. doi:10.1098/rsif.2013.1093

Lewis, P. A. W. (1961). Distribution of the anderson–darling statistic. *Ann. Math. Stat.* 32, 1118–1124. doi:10.1214/aoms/1177704850

Lunn, D. J., Thomas, A., Best, N., and Spiegelhalter, D. (2000). WinBUGS – A Bayesian modelling framework: Concepts, structure, and extensibility. *Statistics and Computing* 10, 325–337. doi:10.1023/A:1008929526011

Marsaglia, G., Tsang, W. W., and Wang, J. (2003). Evaluating kolmogorov's distribution. *Journal of Statistical Software* 8, 1–4. doi:10.18637/jss.v008.i18

Meng, X.-L. (1994). Posterior predictive p-values. *Ann. Stat* 22, 1142–1160. doi:10.1214/aos/1176325622

Metropolis, N., Rosenbluth, A. W., Rosenbluth, M. N., Teller, A., and Teller, E. (1953). Equations of state calculations by fast computing machines. *J. Chem. Phys.* 21, 1087–1091. doi:10.1063/1.1699114

Neal, P. and Roberts, G. (2005). A case study in non-centering for data augmentation: Stochastic epidemics. *Statistics and Computing* 15, 315–327. doi:10.1007/s11222-005-4074-7

O'Neill, P. and Roberts, G. (1999). Bayesian inference for partially observed stochastic epidemics. *J.R. Statist. Soc. A* 162, Part 1, 121–129. doi:10.1111/1467-985X.00125

Papaspiliopoulos, O., Roberts, G., and Skold, M. (2007). A general framework for the parametrization of hierarchical models. *Stat. Sci.* 22, 59–73. doi:10.1214/088342307000000014

Sellke, T. (1983). On the asymptotic distribution of the size of a stochastic epidemic. *J. Appl. Prob* 20, 390–394

Streftaris, G. and Gibson, G. (2012). Non-exponential tolerance to infection in epidemic systems – modelling, inference and assessment. *Biostatistics* 13, 580–593. doi:10.1093/biostatistics/kxs011

Streftaris, G. and Gibson, G. J. (2004). Bayesian inference for stochastic epidemics in closed populations. *Stat Modelling* 4, 63–75. doi:10.1191/1471082X04st065oa

Thomas, A., O'Hara, B., Ligges, U., and Sturtz, S. (2006). Making BUGS Open. *R News* 6, 12–17
